# Circular RNA Telomerase Reverses Endothelial Senescence in Progeria

**DOI:** 10.1111/acel.70021

**Published:** 2025-02-23

**Authors:** Weifeng Qin, Kathrina D. Castillo, Hongye Li, Thi Kim Cuc Nguyen, Daniel L. Kiss, John P. Cooke, Anahita Mojiri

**Affiliations:** ^1^ Department of Cardiovascular sciences Center for Cardiovascular Regeneration, Houston Methodist Research Institute Houston Texas USA; ^2^ Department of Cardiovascular Sciences Center for RNA Therapeutics, Houston Methodist Research Institute Houston Texas USA

**Keywords:** aging, endothelial cells, RNA therapy, senescence, telomerase

## Abstract

Telomeres shorten with each cell division, acting as a chronometer of cell age. The enzyme telomerase, primarily active in stem cells, reverses telomere erosion. We have previously observed that transient transfection with human TERT mRNA extends telomeres and mitigates hallmarks of senescence in replicatively aged human cells or those affected by Hutchinson–Gilford progeroid syndrome (HGPS). However, due to its short half‐life, mRNA requires frequent administration. In this study, we hypothesized that TERT circular (circ) RNA would extend the duration of telomerase expression and be more effective at reversing hallmarks of senescence in endothelial cells derived from HGPS patients. We observe that a single transfection of TERT circRNA is more effective than mRNA in the extension of telomere length, as determined by quantitative fluorescence in situ hybridization. Furthermore, TERT circRNA reduced the number of β‐gal positive cells by three‐fold and normalized nuclear morphology in HGPS endothelial cells (HGPS‐ECs). Moreover, TERT circRNA substantially reduced senescent markers, inflammatory markers, and DNA damage markers, including Progerin, p16, p21, IL‐1B, IL‐6, IL‐8, MCP1, and γH2AX. Additionally, it restored NO production, enhanced cell proliferation, promoted angiogenesis, improved LDL uptake, reduced mitochondrial ROS, and normalized mitochondrial membrane potential more effectively. Our data suggest that TERT circRNA is superior to linear TERT mRNA in reversing processes involved in senescence.

## Graphical abstract

TERT circRNA outperforms linear TERT mRNA in reversing the hallmarks of senescence. TERT circRNA more efficiently restores endothelial function and shows superior capability in enhancing mitochondrial function.

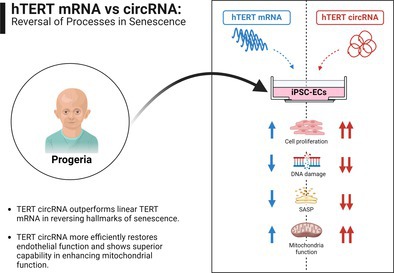

## 1 Introduction

Chronological age is the major risk factor for cardiovascular disease (Dhingra and Vasan 2012). Cardiovascular disease is promoted by processes involved in vascular aging, including endothelial expression of adhesion molecules and chemokines, increased oxidative stress, augmented generation of inflammatory cytokines, as well as reduced vascular generation of homeostatic factors such as endothelium‐derived nitric oxide (Matsushita et al. 2001; Banerjee et al. 2021). We and others have found that processes involved in vascular aging are accelerated in Hutchinson‐Gilford Progeria Syndrome (HGPS). This genetic disorder is caused by a mutation in the lamin A gene (LMNA), leading to the accumulation of a truncated protein called progerin (Lopez‐Otin et al. 2013). Progerin accumulation distorts the nuclear envelope and triggers various cellular alterations associated with aging, including DNA damage, gene expression changes, telomere erosion, and a senescence‐associated secretory phenotype (SASP) (Gorgoulis et al. 2019). Children with HGPS exhibit an accelerated aging phenotype, including sarcopenia, osteoporosis, loss of subcutaneous fat, alopecia, and severe arterial occlusive disease, leading to premature death from myocardial infarction or stroke in their mid‐teens (McClintock et al. 2006). Treatment with a farnesyltransferase inhibitor (Lonafarnib) has shown modest benefits in extending lifespan, highlighting the need for more effective therapies (Capell et al. 2005).

Previously, we have shown that transient transfection with mRNA encoding human telomerase (TERT) is superior to Lonafarnib in reversing the impaired proliferation of HGPS fibroblasts (Li et al. 2017; Li et al. 2019). Subsequently, we used induced pluripotent stem cells (iPSCs) derived from patient‐specific fibroblasts to study vascular disease associated with HGPS. By differentiating these HGPS‐iPSCs into endothelial cells (ECs), we were able to characterize the accelerated aging process of HGPS‐ECs (Matrone et al. 2019). The HGPS‐ECs exhibited the morphological manifestations of senescence with a “fried egg” appearance (a large central nucleus, and rounded cell) and a lobulated nucleus (Capell et al. 2005; Mojiri et al. 2021; Cao et al. 2011). Cell proliferation was severely impaired, and both the level of nitric oxide and the formation of EC networks in Matrigel were reduced. Shortened telomeres were associated with markers of DNA damage and a global disruption of the transcriptional profile with the elaboration of inflammatory cytokines. Treatment with TERT mRNA extended telomere length and increased cell proliferation. Intriguingly, many other manifestations of senescence were also improved with TERT mRNA treatment (Mojiri et al. 2021). Transcriptional profiles of HGPS‐ECs compared to non‐HGPS‐ECs indicated that 1200 genes were differentially expressed, with TERT treatment normalizing the expression of more than 250 genes. Additionally, in a progeria mouse model, treatment with mTERT (delivered by lentivirus) reduced markers of endothelial senescence in vivo and increased lifespan by 20% (Mojiri et al. 2021).

Earlier works have shown that a circular RNA (circRNA) is more stable and provides for longer‐term protein expression (Wesselhoeft et al. 2018). The presumption is that circRNAs have longer half‐lives in vivo because they lack ends that are vulnerable to exonuclease digestion (Wesselhoeft et al. 2018). Because circRNA does not have a traditional capped 5’ end, the translation machinery is recruited, and translation is initiated by an internal ribosome entry site (IRES). In our current study, we tested the hypothesis that a prolonged duration of telomerase expression, provided by the TERT circRNA, would provide for a greater benefit than TERT mRNA. We employed a TERT circRNA with optimal codon sequences to increase both protein output and RNA lifespan (Hanson and Coller 2018; Presnyak et al. 2015). While circRNA is recognized for its potential in promoting prolonged protein expression (Chen et al. 2023), our investigation represents the first cardiovascular application of circRNA. We find that TERT circRNA is superior to TERT mRNA in extending telomeres, normalizing cellular processes, reducing markers of senescence and DNA damage, and restoring mitochondrial function. Importantly, the beneficial effects of TERT circRNA persist over an extended period. To our knowledge, this paper represents the first report of a potentially therapeutic application of a circular RNA to reverse senescence.

## 2 Results

### 2.1 TERT circRNA Is Superior in Restoring HGPS‐ECs Morphology and Function

**FIGURE 1:**
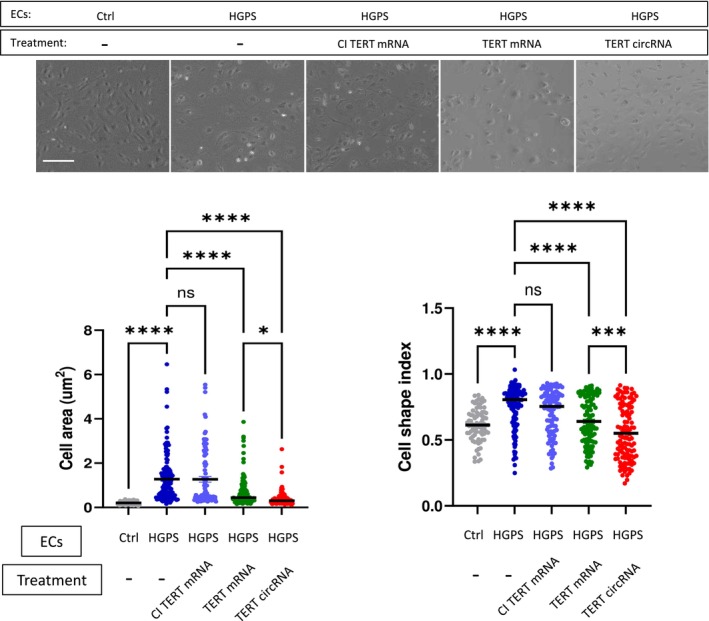
TERT circRNA restores HGPS‐ECs morphology more efficiently than TERT mRNA. Representative images of non‐HGPS‐ECs (Ctrl), HGPS‐ECs, HGPS‐ECs treated with TERT mRNA, catalytically inactive (CI) TERT mRNA, or TERT circRNA are shown. Quantifications of cell area using the polygon tool in ImageJ analysis, and the cell shape index (CSI), defined as (4π × area)/(perimeter^2^) (Tiryaki 2015), show that HGPS‐ECs display a larger and more rounded shape (Mojiri et al. 2021). TERT circRNA was more effective in restoring the cell shape index than TERT mRNA (scale bar: 300 μm, *n* = 79–154). The measurements are expressed as Mean ± Standard Error of Mean (Mean ± SEM). Data between multiple groups were compared by one‐way ANOVA. Results are considered statistically significant with *p* < 0.05(*), *p* < 0.001(***), and *p* < 0.0001(****). Each dot represents one cell from at least three replicates.

To assess the effectiveness of TERT circRNA relative to TERT mRNA, we transfected iPSC‐derived HGPS‐ECs with equal amounts of wild‐type or catalytically inactive (CI) TERT mRNA, as well as TERT circRNA (1 μg RNA/3–4 × 10^5^ cells). We compared the HGPS‐ECs in these conditions to control “non‐HGPS” ECs, which were derived from the genetically normal parents of the children (HGPS is a spontaneous mutation, not present in the genome of the parents). Three to four days post‐transfection, we performed morphometric measurements of the ECs, quantified by cell index values as previously described (Matrone et al. 2019). As expected, HGPS‐ECs were larger and rounder compared to non‐HGPS‐EC controls, consistent with a senescent cell phenotype. Treatment with both TERT mRNA and circRNA improved the morphology of HGPS‐ECs. Notably, the effect of TERT circRNA was more potent than TERT mRNA, as evidenced by a higher number of elongated cells and a smaller total surface area (Figure 1).

**FIGURE 2:**
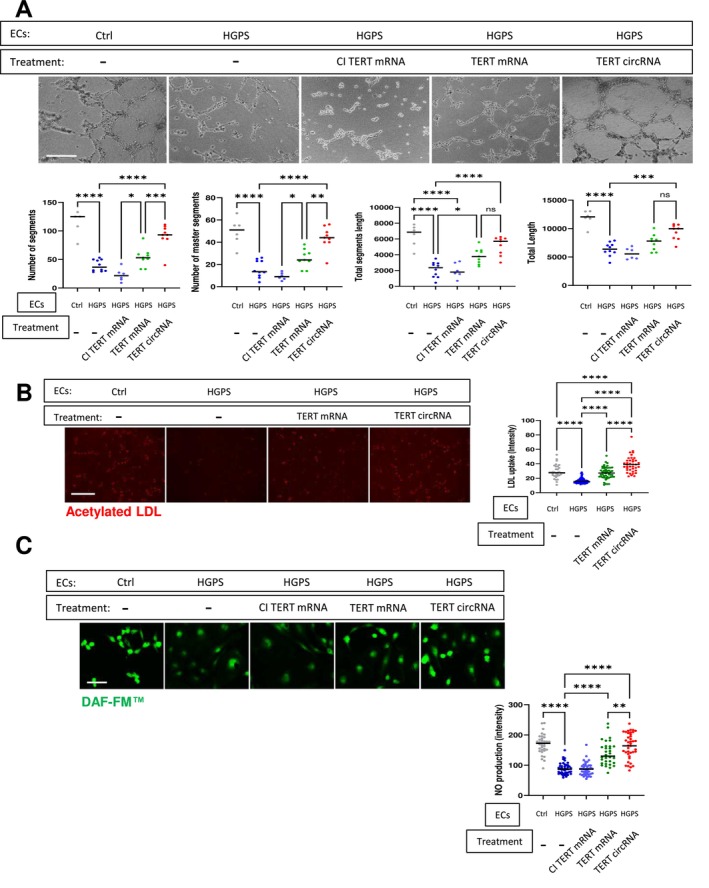
TERT circRNA restores HGPS‐ECs functions more effectively than TERT mRNA. (A) Representative images of Matrigel network segment assays for non‐HGPS‐ECs (Ctrl), HGPS‐ECs, and HGPS‐ECs treated with CI TERT mRNA, WT TERT mRNA, or TERT circRNA are shown. TERT circRNA improved HGPS‐ECs angiogenic processes to a greater degree than those of TERT mRNA (scale bar: 300 μm, *n* = 6). Each dot represents a single field from at least three replicates. The changes between untreated HGPS‐ECs and CI TERT mRNA‐treated cells were not significant. (B) Representative images of acetylated LDL immunofluorescence staining for non‐HGPS‐ECs, HGPS‐ECs, and HGPS‐ECs treated with TERT mRNA or TERT circRNA are shown. TERT circRNA is more effective in improving acetylated LDL uptake (scale bar: 300 μm, *n* = 35–51). Each dot represents one cell from at least three replicates. (C) Representative images of DAF‐FM immunofluorescence staining for non‐HGPS‐ECs, HGPS‐ECs, and HGPS‐ECs treated with TERT mRNA, CI TERT mRNA, or TERT circRNA are shown (scale bar: 150 μm, *n* = 32–44). Each dot represents one cell from at least three replicates. Data between multiple groups were compared by one‐way ANOVA. Results are considered statistically significant with *p* < 0.05(*), *p* < 0.01(**), *p* < 0.001(***), and *p* < 0.0001(****).

We then compared the effect of TERT circRNA vs. TERT mRNA on EC‐specific functions. Treatment with a single dose of TERT circRNA (1 μg/mL) improved HGPS‐ECs capacity for network formation in Matrigel, as indicated by the number of segments, master segments, and tube length, to a greater degree than TERT mRNA (Figure 2A). Treatment with TERT circRNA was also superior with respect to enhancing the uptake of acetylated LDL (ac‐LDL) and the generation of nitric oxide (Figure 2B,C). Notably, CI TERT, which lacks the catalytic activity of telomerase, did not exert any beneficial effects, consistent with the notion that the catalytic activity of telomerase is necessary for these benefits (Li et al. 2017; Li et al. 2019).

### 2.2 TERT circRNA Reduces Senescence‐Associated β‐Galactosidase (SA‐β‐Gal) in a Dose‐Dependent Manner

**FIGURE 3:**
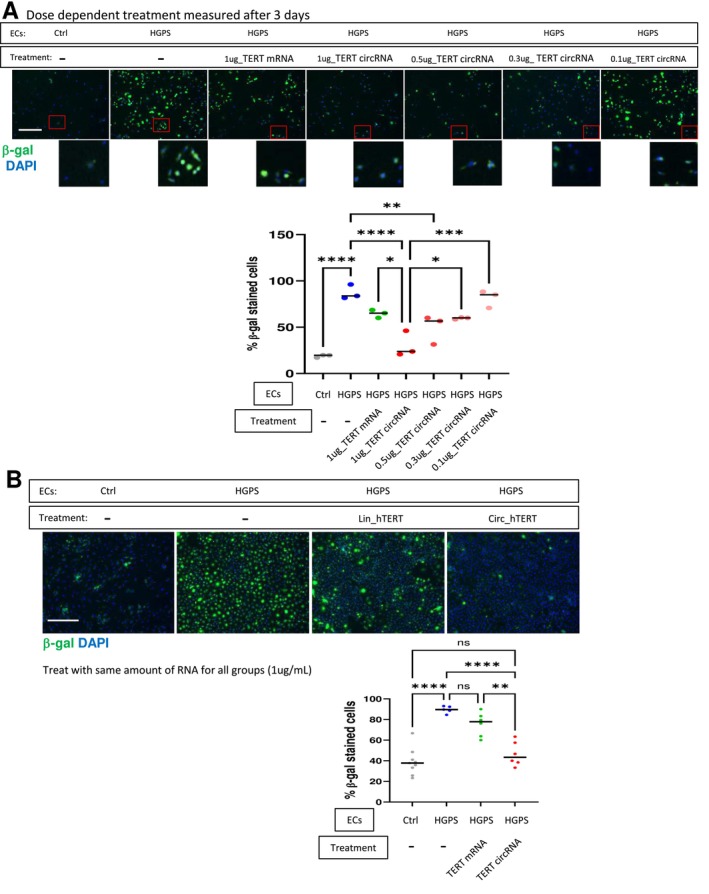
TERT circRNA reduces senescence‐associated β‐galactosidase to a greater degree. (A) Representative images of β‐galactosidase immunofluorescence staining for HGPS‐ECs 3 days after treatments with 1 μg/mL TERT mRNA and different dosages of TERT circRNA, including 1, 0.5, 0.3, and 0.1 μg/mL (scale bar: 300 μm). Insets are provided for better visualization of nuclei. Quantification of the percentage of β‐galactosidase‐positive cells in cells treated with TERT circRNA or TERT mRNA (*n* = 3). (B) Representative images of β‐galactosidase immunofluorescence staining are shown (scale bar: 300 μm). Quantification of β‐galactosidase staining of cells treated with 1 ug/mL TERT circRNA or TERT mRNA over a 28‐day culture period (*n* = 5–9). The data are expressed as Mean ± SEM. Data between multiple groups were compared by one‐way ANOVA. Results are considered statistically significant with *p* < 0.05(*), *p* < 0.01(**), *p* < 0.001(***), and *p* < 0.0001(****). Each dot represents one field from at least three replicates.

SA‐β‐gal is a well‐described marker of senescent cells (Debacq‐Chainiaux et al. 2009). We compared the dose‐dependent effects of TERT mRNA vs. circRNA on the reduction of SA‐β‐gal, 3 or 28 days after a single transfection. When compared to non‐HGPS‐EC controls, we consistently observed high levels of β‐gal positive cells in HGPS‐ECs. We observed a dose‐dependent reduction in β‐gal positive cells with TERT RNA treatment; however, the circRNA treatment resulted in a greater effect than that achieved with TERT mRNA (Figure 3A). Notably, a dose‐dependent effect of TERT circRNA was also observed in the normalization of nuclear morphology when more than 200 nuclei were quantified in a blinded manner (Figure S1).

In addition, the effect of TERT circRNA was more prolonged than that of TERT mRNA. In the HGPS‐ECs treated with TERT circRNA, the number of β‐gal positive cells remained low and comparable to that of non‐HGPS‐ECs at 28 days post transfection. However, in the HGPS‐ECs treated with TERT mRNA, the benefit was attenuated after 28 days, and the number of β‐gal positive cells was no longer different from untreated HGPS‐ECs (Figure 3B). Restoration of telomere length was maintained 28 days after TERT circRNA treatment in HGPS‐ECs and was greater than that observed with TERT mRNA (Figure S2). These data show that TERT circRNA is more potent in reversing processes associated with senescence and provides a more durable response than TERT mRNA.

### 2.3 TERT circRNA More Effectively Reduces the Expression of Senescence and Inflammatory Genes

**FIGURE 4:**
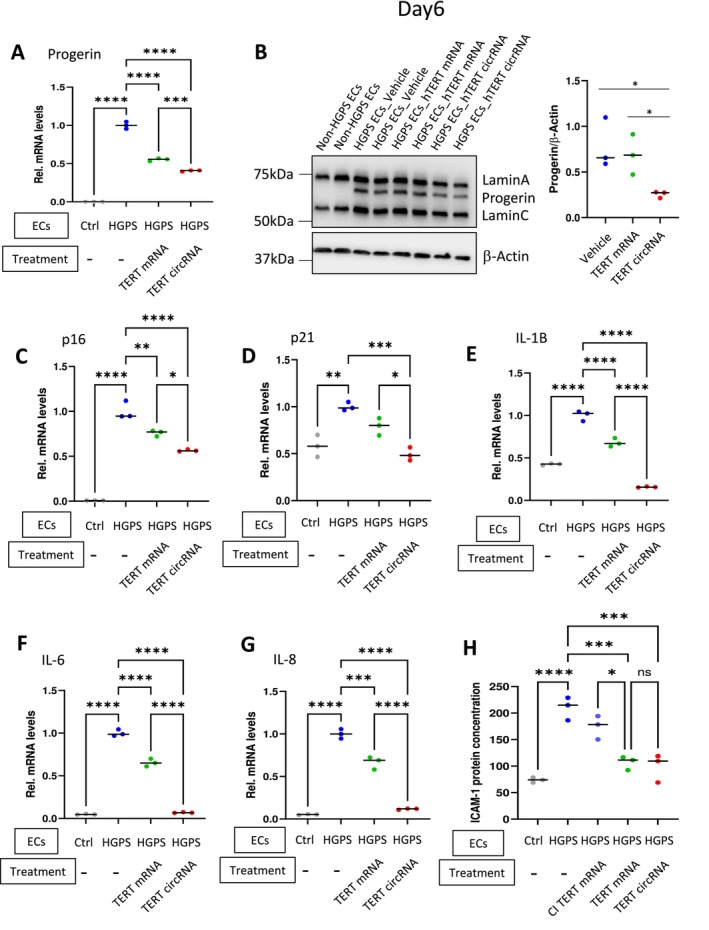
TERT circRNA more effectively reduces expression of genes involved in senescence and inflammation in HGPS‐ECs. (A) Progerin expression at mRNA level 2 days after transfection (*n* = 3). (B) Representative Western blot for progerin expression at day 6 after treatment. Quantification is shown for day 6. (C‐G), Real‐time PCR analysis examining relative levels of p16, p21, IL‐1B, IL‐6, and IL‐8 mRNAs (*n* = 3). (H), ELISA analysis on collected conditioned media after 4 h of transfection with different TERT RNAs (*n* = 3). The data are expressed as Mean ± SEM. Data between multiple groups were compared by one‐way ANOVA. Results are considered statistically significant with *p* < 0.05(*), *p* < 0.01(**), *p* < 0.001(***), and *p* < 0.0001(****). Each dot represents one replicate.

Previously, we showed a significant reduction of progerin expression in HGPS‐ECs after two consecutive treatments with TERT mRNA (Mojiri et al. 2021). Our new data show a greater reduction in progerin levels when HGPS‐ECs were treated with TERT circRNA by comparison to TERT mRNA at day 6 (Figure 4A,B). Furthermore, TERT circRNA was also more effective in reducing two cell cycle arrest genes, p16 and p21, as well as proinflammatory markers IL1b, IL6, and IL8 in HGPS‐ECs (Figure 4C–G). Both TERT mRNA and circRNA reduced ICAM‐1 secretion into the media to a similar degree (Figure 4H). However, the preponderance of the data indicates that TERT circRNA is more effective than TERT mRNA in reducing the expression of genes involved in senescence and/or inflammation.

### 2.4 TERT circRNA More Effectively Increases Proliferation and Reduces DNA Damage Markers

**FIGURE 5:**
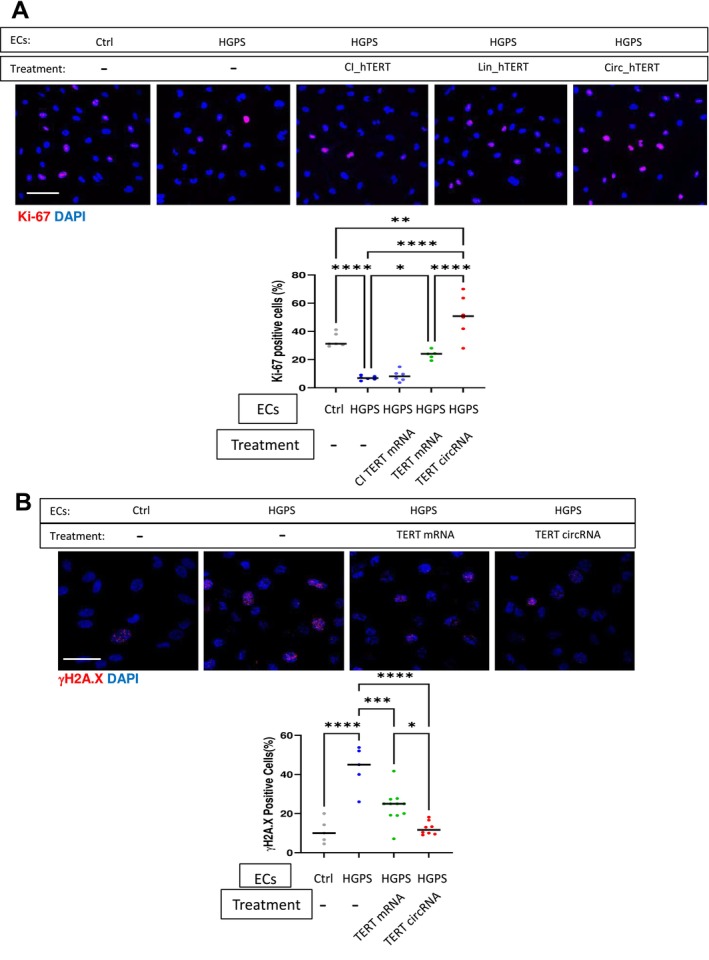
TERT circRNA increases proliferative markers and reduces DNA damage markers more effectively than TERT mRNA. (A) Representative images of Ki‐67 immunofluorescence staining for non‐HGPS‐ECs (Ctrl), HGPS‐ECs, and HGPS‐ECs treated with TERT mRNA, CI TERT RNA, or TERT circRNA are shown. Quantification of the percentage of Ki‐67 positive cells (*n* = 6). 30–40 cells were counted in each field of view. (B) Representative images of γH2AX immunofluorescence staining for non‐HGPS‐ECs, HGPS‐ECs, and HGPS‐ECs treated with TERT mRNA, CI TERT RNA, or TERT mRNA are shown (scale bar: 50 μm). Representative figures and quantification of γH2AX immunofluorescence staining (scale bar: 75 μm) (*n* = 5). Data between multiple groups were compared by one‐way ANOVA. Results are considered statistically significant with *p* < 0.05(*), *p* < 0.01(**), and *p* < 0.0001(****). Each dot represents one field from at least three replicates.

HGPS‐ECs exhibit a poor growth rate and an accumulation of DNA damage markers (Xu et al. 2022). Our earlier work demonstrated that two transfections with TERT mRNA can restore the growth rate of HGPS‐ECs and reduce the DNA damage marker γH2A.X (Mojiri et al. 2021). Interestingly, it appears that a single transfection with TERT mRNA increases Ki‐67 expression, suggesting a higher proliferative rate (Andres‐Sanchez et al. 2022), but the reduction of γH2A.X was not significant within 3–4 days after transfection. However, just one transfection with TERT circRNA was sufficient to increase the number of Ki‐67‐positive cells and reduce γH2A.X levels (Figure 5A,B).

### 2.5 TERT circRNA Restores Mitochondrial Function More Effectively

**FIGURE 6:**
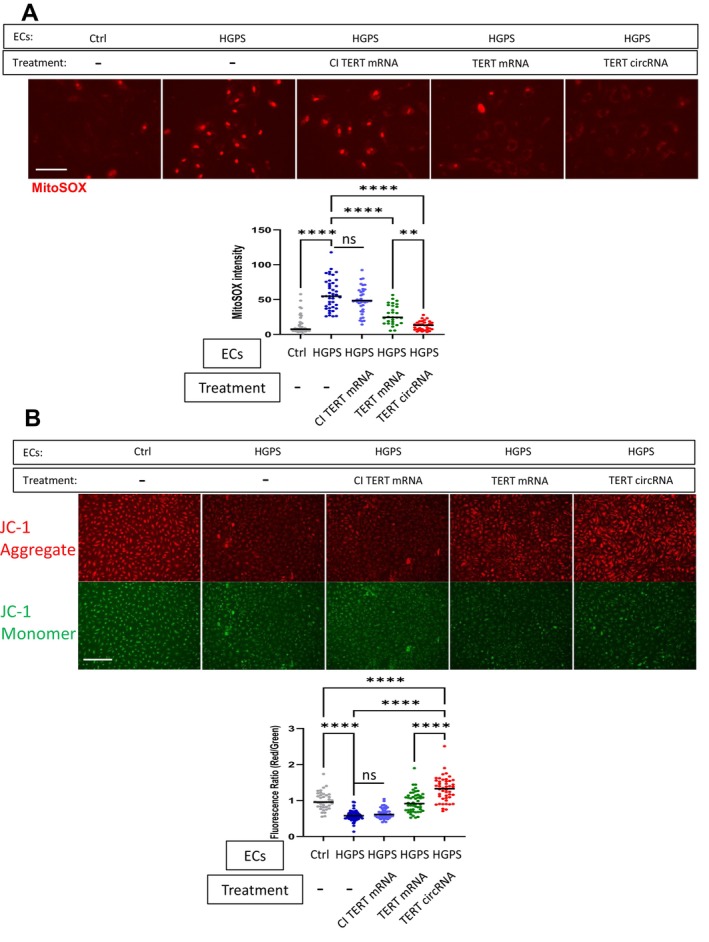
TERT circRNA rescues mitochondrial functions more effectively. (A) Representative images of MitoSOX staining for non‐HGPS‐ECs (Ctrl), HGPS‐ECs, and HGPS‐ECs treated with TERT mRNA, CI TERT RNA, or TERT circRNA are shown (scale bar: 150 μm). Quantification of MitoSOX staining. (B) Representative images of JC‐1 staining for non‐HGPS‐ECs, HGPS‐ECs, and HGPS‐ECs treated with TERT mRNA, CI TERT RNA, or TERT mRNA are shown (scale bar: 300 μm). Quantification of JC‐1 staining (*n* = 38–65). Data between multiple groups were compared by one‐way ANOVA. Data Results were considered statistically significant with *p* < 0.01(**), and *p* < 0.0001(****). Each dot represents one cell from at least three replicates.

Telomerase is known to play a role in normal mitochondrial function (Ahmed et al. 2008). To understand whether transient transfection with TERT could restore mitochondrial function in HGPS‐ECs, we transfected HGPS‐ECs with either CI or WT TERT mRNA, or TERT circRNA (1 μg/mL in each case). We used MitoSOX to detect mitochondrial superoxide as an indicator of mitochondrial dysfunction. MitoSOX fluorescence is increased with oxidative stress due to increased reactive oxygen species (ROS) production or impaired antioxidant defenses (Hao et al. 2022). We found that both TERT mRNA and TERT circRNA reduced MitoSOX fluorescence (Figure 6A), but the level of recovery was greater in HGPS‐ECs treated with TERT circRNA. As an orthogonal approach to assess mitochondrial function, we also measured mitochondrial membrane potential using JC‐1 dye. JC‐1 dye exhibits potential‐dependent accumulation in mitochondria, characterized by a green fluorescence when in its monomeric form. This fluorescence shifts to red as the dye forms red fluorescent J‐aggregates in a concentration‐dependent manner. A decrease in the red/green fluorescence intensity ratio indicates mitochondrial depolarization, suggesting mitochondrial dysfunction. Treatment with TERT mRNA or circRNA enhanced membrane potential in HGPS cells, but TERT circRNA was more effective (Figure 6B). The use of CI TERT did not improve mitochondrial oxidative stress or membrane potential.

### 2.6 Beneficial Effects of TERT circRNA Attributed to Its Circular Structure Rather Than Its Optimal Codon Sequences

**FIGURE 7:**
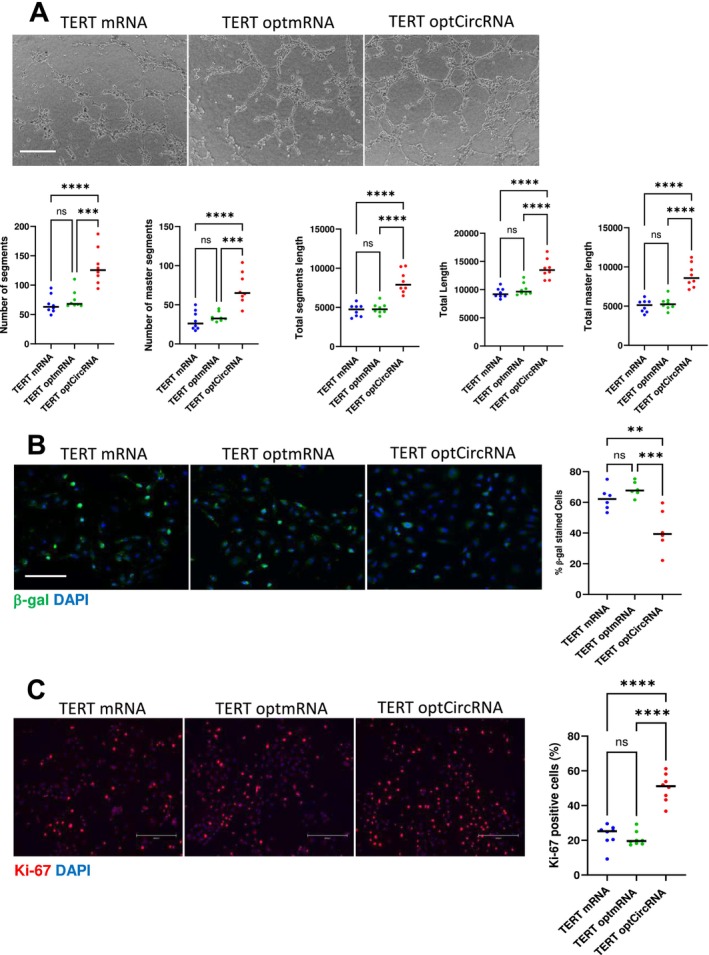
Beneficial effects of TERT RNA attributed to its circular structure rather than its optimal codon sequences. (A) Representative images of Matrigel network segment assays for HGPS‐ECs treated with TERT mRNA, codon‐optimized TERT mRNA, or codon‐optimized TERT circRNA 48 h post‐transfection are shown (scale bar: 300 μm). Quantification of Matrigel network segment assays (*n* = 8). (B) Representative images of β‐galactosidase immunofluorescence staining for HGPS‐ECs treated with TERT mRNA, optimized TERT mRNA, or TERT circRNA are shown (scale bar: 300 μm). Quantification of the percentage of β‐galactosidase positive cells (*n* = 6). (C) Representative images of Ki‐67 immunofluorescence staining for HGPS‐ECs treated with TERT mRNA, codon‐optimized TERT mRNA, or TERT circRNA are shown (scale bar: 300 μm). Quantification of Ki‐67 immunofluorescence staining A (*n* = 8). Data between multiple groups were compared by one‐way ANOVA. Data Results were considered statistically significant with *p* < 0.01(**), *p* < 0.001(***), and *p* < 0.0001(****). Each dot represents one field from at least three replicates.

The TERT circRNA is generated (Figure S3) using a novel codon optimality formula that improves the translational output of each RNA, thereby increasing the expression of the encoded protein (Hanson and Coller 2018). The integrity of the optimal TERT RNAs was validated by agarose gel and Tapestation assays, and the coding ability was verified via western blotting (Figure S3). The optimal sequence also increases the stability of the RNA (Presnyak et al. 2015). To identify whether the codon optimality formula was solely responsible for the greater effects of TERT circRNA, we generated TERT mRNA utilizing the same codon optimality formula. We transfected HGPS‐ECs with either original TERT mRNA, TERT mRNA with optimal codons, or TERT circRNA. We observed no difference in network formation when using TERT mRNA with its original sequence or with the sequence modified by the optimality formula (Figure 7A,B). Additionally, both RNAs had an equivalent effect to reduce β‐gal positive cells and to increase Ki‐67 expression. The efficacy of TERT circRNA in reversing markers of senescence was greater than that of TERT mRNA (Figure 7A,B). These data suggest that the beneficial effects of TERT circRNA are primarily due to its circular structure rather than the optimal codons used to generate it.

**FIGURE 8:**
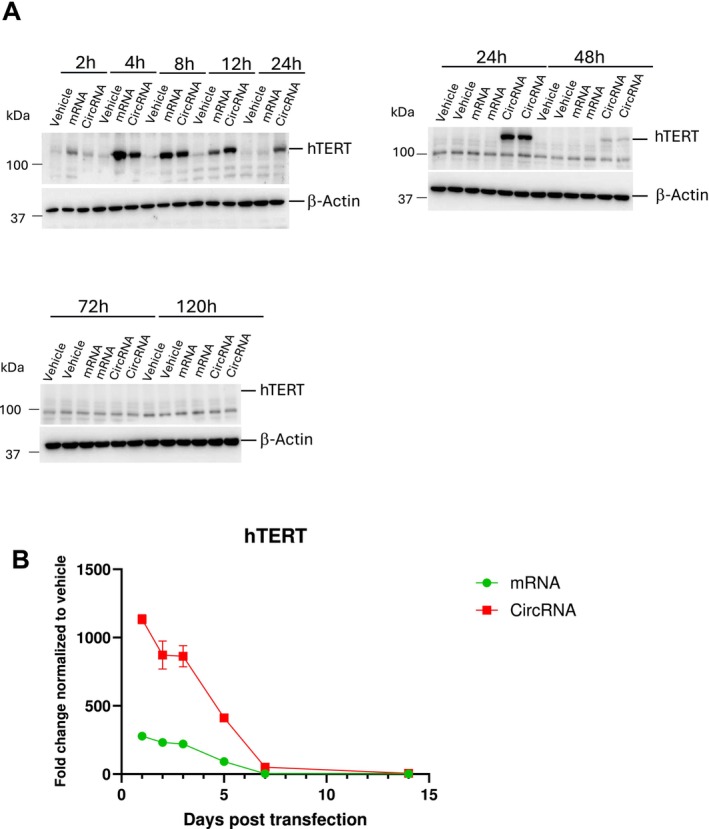
Telomerase protein expression is sustained longer after transfection with TERT circRNA when compared to TERT mRNA. (A) Representative Western blot for hTERT expression at indicated time points after transfection with TERT mRNA, circRNA or GFP Mrna (1 μg/mL) as a control in vehicle group. (B) Real‐time PCR analysis shows the fold change in hTERT expression at the mRNA level, normalized to endogenous TERT, at specified time points following transfection with TERT mRNA, circRNA or GFP mRNA as a control in vehicle group.

Additionally, we measured TERT protein and RNA levels over time to compare the half‐lives of TERT in linear vs. circular RNA in HGPS‐ECs (Figure 8A). Our data show that at the protein level, within 2 h post‐transfection, a higher amount of protein is generated from TERT mRNA compared to circRNA, which also shows an increase at 4 h. However, by ~8 h post‐transfection, the amounts of TERT protein produced from both mRNA and circRNA is relatively equal. Notably, the level of protein generated from circRNA remains high at 12, 24, and 48 h post‐transfection, whereas the protein from TERT mRNA decreases at 12 h and is essentially undetectable by 24 h. At the RNA level, high levels of circRNAs were consistently detected for over 120 h after transfection (Figure 8B). Together, these data show that TERT circRNA has greater longevity and more persistent translational activity than TERT mRNA.

## 3 Discussion

The primary cause of morbidity and mortality in children with HGPS is vascular disease (Taimen et al. 2009). The endothelium plays a crucial role in vascular homeostasis, making endothelium‐targeted strategies sensible for these children (Xu et al. 2022; Nazari‐Shafti and Cooke 2015). Previously, we demonstrated that two consecutive transfections of TERT mRNA can partially restore the function and morphology of HGPS‐ECs (Mojiri et al. 2021; Ramunas et al. 2015). TERT mRNA treatment increased telomere length, improved cellular and nuclear morphology, and enhanced cell proliferation and function (Mojiri et al. 2021; Ramunas et al. 2015). Furthermore, transcriptome profile analysis revealed that of the 1200 genes differentially expressed between control ECs and HGPS‐ECs, the expression of more than 250 genes was normalized after TERT mRNA therapy, with many others showing a trend toward recovery (Mojiri et al. 2021; Ramunas et al. 2015).

We have optimized our TERT mRNA to enhance stability and to reduce immunogenicity by using modified nucleosides and minimizing impurities of IVT synthesis such as dsRNA that can activate toll‐like receptors (Kariko et al. 2005; Scumpia et al. 2005). However, due to the short lifespan of the mRNA in vivo, two sequential transfections were needed to achieve recovery of morphology and function in HGPS‐ECs. The liposomal transfection medium also has some cellular toxicity, and subjecting HGPS‐ECs to two liposomal transfections can further compromise these fragile cells. Similarly, the lipid nanoparticles that might be used to treat patients are known to have some hepatotoxicity with repeated doses (Lv et al. 2006; Hou et al. 2021). Therefore, to reduce the number of treatments required to have a beneficial effect, we developed TERT circRNA with increased stability and prolonged duration of expression. Circular RNAs escape rapid degradation by exonucleases, which primarily degrade mRNAs after deadenylation and decapping (Fumagalli et al. 2012). Additionally, the circRNAs contain an IRES sequence required for ribosomal detection and translation (Wesselhoeft et al. 2018; Chen et al. 2023). Due to their longer stability within the cell, circRNAs produce more protein over an extended period, reducing the need for repeated dosing. Notably, circRNAs do not require modified nucleosides to escape innate immune activation, thereby also reducing the cost of goods. Furthermore, one group reported data consistent with circRNAs being more thermostable, improving stability during storage (Zhao et al. 2021).

In our current study, we demonstrated for the first time the potent effect of TERT circRNA compared to TERT mRNA. This includes greater reductions in progerin levels, DNA damage, and proinflammatory markers when equal masses of the two forms of TERT RNAs are transfected (Figures 1, 2, 3, 4).

By comparison to TERT mRNA, TERT circRNA persists longer and extends protein expression, as observed in Figure 8. The gradual increase in TERT protein generation over time from TERT circRNA contrasts with the immediate increase in TERT proteins from TERT mRNA. This difference is likely because cap‐dependent translation initiation on linear mRNAs is thought to be more efficient than IRES‐driven translation of circRNAs.A (Chen et al. 2023). Notably, in Figure 8, our western blots only detect TERT protein for ~48 h, but our qPCR assays consistently detect TERT circRNA for three times that duration. This discrepancy is likely caused by the different sensitivities of the assays, with qPCR's amplification steps providing far greater sensitivity than the antibody‐based detection used in the western blots.

A role for telomerase in maintaining mitochondrial function has been suggested (Haendeler et al. 2009), and our study is consistent with these reports (Ahmed et al. 2008; Haendeler et al. 2009). Both mRNA and circRNA forms of TERT improve mitochondrial function, as evidenced by dyes sensitive to membrane potential or ROS concentration.

Notably, TERT circRNA is more effective in restoring mitochondrial function than TERT mRNA (Figure 6). As both RNA forms contain signal sequences that direct TERT to the nuclei or mitochondria, this superior efficacy is likely caused by the longer persistence of telomerase protein in cells, rather than changes in TERT cellular distribution (Niu et al. 2023). Although mitochondrial DNA does not contain telomere sequences, an active form of TERT is required for its beneficial mitochondrial effects (Niu et al. 2023). We observed no significant changes in HGPS‐ECs treated with CI TERT mRNA, suggesting that these non‐canonical activities are mediated by the reverse transcriptase activity of TERT. It has been reported that aging and stress can increase nuclear ROS (Rajapakse et al. 2011; Storz 2006; Kim and Lee 2024). Consistently, we have observed a nuclear MitoSox signal in HGPS cells, which may be related to nuclear lobulation and loss of nuclear membrane integrity. Furthermore, we observed that TERT circRNA was more effective than TERT mRNA in reducing the nuclear MitoSox signal and in restoring nuclear morphology.

Our data indicates that the enhanced ability of TERT circRNA to restore and sustain EC function is largely due to the circular nature of TERT circRNA, rather than the codon optimality formula employed. This finding aligns with recent studies showing that VEGF‐A circRNA enhances wound healing due to the sustained expression and ongoing release of VEGF‐A, suggesting potential applications for the treatment of diabetic foot ulcer wounds (Liu et al. 2024). Previously, we demonstrated the benefits of TERT mRNA in reversing senescence markers in HGPS‐ECs; however, its use is limited by its susceptibility to degradation, poor stability, and the need for multiple transfections. In contrast, TERT circRNA exhibits remarkable stability due to its covalently closed ring structure, which shields it from exonuclease‐mediated degradation (Welden and Stamm 2019). Supporting this, our data reveal that TERT circRNA provides greater and more prolonged expression of the encoded protein and leads to superior restoration of HGPS‐ECs morphology and function. Furthermore, TERT circRNA outperforms TERT mRNA by halting the accumulation of senescence markers and enhancing ECs functions, including mitochondrial function. Further translational studies in mouse models using TERT circRNA in the context of accelerated aging could provide insights into potential telomerase‐based therapies for age‐related diseases and conditions associated with accelerated aging.

## 4 Methods and Materials

### 4.1 Maintenance of Human Induced Pluripotent Stem Cell

Human iPSC lines (HGFDFN168 iPSC1P, HGADFN167 iPSC 1Q, HGPS‐F143 iPSC 1C, HGMDFN090 iPSC 1B) were provided by the Progeria Research Foundation Cell and Tissue Bank and were cultured and maintained on Matrigel (BD Biosciences)‐coated plates (Corning) in mTeSR plus medium (STEMCELL Technologies) according to protocol and our previous work (Mojiri et al. 2021). The iPSCs were passaged upon 70% confluency and maintained in humidified incubators at 37°C and 5% CO_2_ (Thermo Scientific).

### 4.2 Generation of Endothelial Cells From Induced Pluripotent Stem Cell (iPSC)

Methods to generate ECs from iPSCs were adopted from our previously established protocol (Matrone et al. 2019; Mojiri et al. 2021). Briefly, when the iPSCs reached 60% confluence, they were incubated with DMEM/F12 medium supplemented with four factors: Wnt agonist CHIR 99021 (5 mM, Selleck), bone morphogenetic protein‐4 (BMP4, 25 ng/mL, Peprotech), B27 supplement (Thermo Fisher Scientific), and N2 supplement (Thermo Fisher Scientific). Cells were detached using accutase on day 3 and seeded onto Matrigel‐coated plates. They were then incubated with StemPro medium supplemented with forskolin (5 Μm, LC Laboratories), VEGF (5 ng/mL, Peprotech), and polyvinyl alcohol (2 mg/mL, Sigma). The medium was changed to complete endothelial growth medium (EGM‐2MV, Lonza) on day 7. By day 11, ECs were isolated using CD31 microbeads or FACS, employing both CD31 and CD144. The isolated iPSC‐ECs were cultured and used for subsequent experiments between days 12 and 14.

### 4.3 Cell Treatment With TERT RNA

When HGPS‐ECs reached 70%–80% confluence, transfection with different TERT RNAs was performed using the JetMessenger reagent (Polyplus), following the manufacturer's protocol. Briefly, 2 μg of RNA was added to 200 μL of mRNA buffer, followed by 4 μL of transfection reagent. The mixture was incubated at room temperature for 15 min after vortexing. A final concentration of 1 μg/mL or the indicated concentration of RNA was added to the cells. The cells were washed twice with PBS after 4 h of transfection. Unless otherwise specified, experiments were performed 48 h after transfection.

### 4.4 Measurement of Nitric Oxide Production

48 h post transfection with different TERT RNAs, iPSC‐ECs were detached with trypsin and seeded onto a 24‐well plate for another 48 h to reach 80%–90% confluence. DAF‐FM DA fluorescent dye (Cat# D‐23841, Invitrogen) was used to measure intracellular nitric oxide as earlier (Mojiri et al. 2021). Briefly, ECs were incubated with 5 μM DAF‐FM DA for 30 min at 4°C after being washed twice with PBS. The dye was washed away after incubation, and endothelial growth medium was added to allow the complete de‐esterification of AM moieties and nitric oxide generation. Images were taken using a 20X fluorescence microscope (EVOS M5000) and analyzed with ImageJ. Signal intensity was plotted as the mean fluorescent signal in each cell.

### 4.5 Dil‐Ac‐LDL Uptake Assay

48 h post‐treatment with different TERT RNAs, iPSC‐ECs were detached with trypsin and seeded onto a 24‐well plate for another 48 h to reach 30%–40% confluence. As previously, acetylated low‐density lipoprotein (Ac‐LDL) dye was used to assess the LDL uptake ability of the cells (Mojiri et al. 2021). Briefly, ECs were starved using basal endothelial growth medium supplemented with 0.3% BSA for 24 h. A working solution of ac‐LDL with a concentration of 10 μg/mL was added to the cells for a 2‐h incubation at 37°C. After washing twice with 0.3% BSA, images were taken under a fluorescence microscope and then analyzed with ImageJ.

### 4.6 β‐Gal Staining Protocol

β‐Gal staining was performed according to the manufacturer's protocol (CellEvent Senescence Green Detection Kit. Lot: 2549282). In brief, cells were harvested using trypsin enzyme, resuspended in 1X PBS, and followed by fixation in 4% paraformaldehyde for 10 min at room temperature. After washing, cells were then stained with the CellEvent Senescence Green Probe (dilution: 1/1000 in CellEvent Senescence Buffer) for 90 min in a 37°C incubator without CO_2_. Images were acquired by confocal FV3000.

### 4.7 Bio‐Rad ELISA Analysis

The concentrations of cytokines were measured using Bioplex ProTM human cytokine (Bio‐Rad Laboratory, Hercules, CA, USA) according to the manufacturer's protocol. In brief, EC media was collected and then probed for 2 markers of inflammation, soluble ICAM‐1 and VCAM‐1. Beads were washed 3 times with washing buffer and were incubated for 1 h with fluorescent secondary antibodies of different colors. Finally, the beads were washed, the final buffer was added, and the plates were read (region for ICAM‐1 is 12) to detect inflammatory markers using the Bio‐Rad detection machine (Luminex 200 System).

### 4.8 Angiogenesis Assay

Vascular network formation assays were performed as previously described (Matrone et al. 2019; Mojiri et al. 2021). ECs treated with different TERT RNAs, then 48 h post treatment, cells were seeded in growth factor‐reduced Matrigel and incubated with EGM‐2 MV medium at a density of 45–50,000 cells per well in a 48‐well plate. Images were taken after 4 h in three random microscopic fields per well.

### 4.9 Synthesis of TERT circRNA

The template plasmid was linearized by restriction digest and purified using a DNA Clean & Concentrator‐25 kit (Cat# D4033, Zymo Research). 1 μg of linearized plasmid DNA was used as a template for 20 μL in vitro transcription reactions using the HiScribe T7 High Yield RNA Synthesis Kit (Cat# E2040S, NEB) following the manufacturer's suggested protocol. Reactions were incubated for 2 h and 30 min at 37°C with shaking at 210 rpm and the RNA product was column purified using the Monarch RNA Cleanup Kit (Cat# T2050S, NEB). Circular RNA synthesis was carried out as described elsewhere with modifications below (Aymard et al. 2014). To initiate circularization, 100 μg of RNA precursor was incubated in circularization buffer (50 mM Tris PH 7.5, 1 mM DTT, 2 mM rGTP, 10 mM MgCl_2_) in a 100 μL reaction at 55°C for 5 min. The circularization reaction was column purified using a Monarch RNA Cleanup Kit (NEB). To remove RNA contaminants, RNA was treated with 1 unit of RNase R (Cat# RNR07250, Biosearch Technologies) per microgram of RNA for 1 h at 37°C. Following guanidinium‐thiocyanate‐phenol (TriReagent, Cat# R2050‐1‐200, Zymo Research) and chloroform extraction, the upper aqueous phase was column‐purified using the Monarch RNA Cleanup Kit (NEB) according to the manufacturer's instructions. Sample concentration and purity were determined using a DeNovix spectrophotometer, and lots of circRNA were verified (for intactness of the circRNA and low levels of mRNA contaminants) using a 1.2% FlashGel RNA cassette (Cat# 57027, Lonza) and using an Agilent TapeStation following the manufacturer's protocol for RNAs.

### 4.10 Quantitative Fluorescence In Situ Hybridization (q‐FISH)

q‐FISH experiments were performed according to our previously established protocol (Mojiri et al. 2021). In brief, using colcemid (Roche) treatment, cells were arrested in metaphase and then collected into a 15 mL microcentrifuge tube. 4 mL of 37°C warmed hypotonic solution (50 nM KCl, Sigma‐Aldrich) was added dropwise. A 3:1 ethanol/acetic acid mixture (Sigma‐Aldrich) was added while vortexing. The cells were centrifuged at 1000 rpm for 5 min, dropped onto a glass slide, and fixed in 3.7% formaldehyde, followed by dehydration with an ethanol series: 5 min each in 70%, 85%, and 100%. The telomere probe (PNA Bio F1013 TelC‐Alexa 647) was added to the slide and incubated overnight at 4°C. After washing twice with 70% formamide and twice with 1% BAS, respectively, the slides were dehydrated with ethanol. DAPI was added, and telomere length was reported as the intensity of signals normalized to the DAPI signal.

### 4.11 qPCR Analysis

Cell pellets are collected and total RNA was extracted using an RNeasy Mini Kit (QIAGEN) according to the manufacturer's protocol. cDNA was synthesized from the 100 ng extracted RNA for each reaction using 5x iScript Reverse Transcriptase Supermix according to the manufacturer's instructions (Bio‐Rad Laboratories Inc.). A volume of 10 μL was used for each qPCR reaction: 5 μL of Power SYBR Green Master Mix (Applied Biosystems, Thermo Fisher Scientific), 0.5 μL of primers (reverse and forward), 1 μL of cDNA, and 3.5 μL of nuclease‐free water were mixed in PCR tubes. Quantitative real‐time PCR was performed using a QuantStudio 12 K Flex (Applied Biosystems, Life Technologies). Results were reported as mRNA levels relative to control samples by the ΔΔCt calculation method (Rao et al. 2013).

### 4.12 Immunofluorescence Staining

Beta‐gal staining (Cat# 66586‐1‐Ig), Ki‐67 staining (Cat# MA5‐14520), and LaminA (Cat# MA1‐06101) and γH2A.X staining (Cat# JBW301) were performed using modifications of an earlier protocol (Mojiri et al. 2021). Briefly, cells were cultured on chamber slides and then fixed with 4% paraformaldehyde in PBS at room temperature for 10 min. Permeabilization was conducted by incubating the samples with 0.15% Triton X‐100 in 3% BSA in PBS for 30 min. The samples were washed three times with PBS, each time for 5 min. Blocking was conducted by incubating the samples with 3% BSA in PBS for another 30 min. The samples were then incubated with primary antibodies diluted in 3% BSA in PBS overnight in a 4°C humidified chamber. Subsequently, the samples were incubated with secondary antibody diluted in 3% BSA for 2 h at room temperature in the dark. Finally, 50ul of mounting medium with DAPI was added to each sample. The samples were then covered with a coverslip and sealed with nail polish to prevent drying and movement.

### 4.13 MitoSOX Detection Assay

The MitoSOX (Cat# M36008) reagent was used to detect mitochondrial ROS according to the manufacturer's protocol. In brief, the 5 mM MitoSOX reagent stock solution was diluted in HBSS buffer (provided by the kit) to make a 5 μM solution. Cells prepared in a 24‐well plate were incubated with the 5 μM MitoSOX reagent working solution for 10 min at 37°C, protected from light. After washing with warm PBS, the cells were imaged under a fluorescent microscope.

### 4.14 JC‐1 Staining

JC‐1 staining was performed to evaluate mitochondrial membrane potential, following the manufacturer's protocol (Cat# ENZ‐51018). In brief, ECs were cultured in a 24‐well plate after treatment with different TERT RNAs. The cells were washed with ice‐cold PBS and incubated with JC‐1 dye for 20 min at 37°C, protected from light. The cells were then washed to remove any free dye in the supernatant. Images were taken under a 10x magnification fluorescence microscope. The JC‐1 monomer (green fluorescence) is more prevalent in mitochondria with a low membrane potential (MMP), whereas the JC‐1 aggregate (red fluorescence) is more prominent in mitochondria with a higher MMP.

### 4.15 Validating Protein Expression From Optimal TERT RNA

To verify protein expression from the newly designed optimal TERT RNA sequence, U2OS cells (1 × 10^6^ cells) were transfected with 2 μg of optimal TERT RNA or TERT circRNA. Non‐transfected cells served as a negative control. Cells were harvested 24 h post transfection, and proteins were extracted using the Mammalian Protein Extraction Reagent, M‐PER (Thermo Scientific, Cat. No. 78501). Protein concentration was determined using the DeNovix spectrophotometer, and protein lysates (50 μg) were analyzed by western blotting (Berger et al. 2019).

### 4.16 Western Blotting

Equal amounts of total protein were resolved on SDS‐PAGE gels (BioRad) and blotted to TransBlot Turbo PVDF membranes using the Trans‐Blot Turbo system (BioRad) following the manufacturer's instructions. TERT levels were detected using rabbit polyclonal anti‐TERT antibody (Rockland, Cat. No. 600–401‐252) diluted 1:500 in 5% BSA in 1X TBST. Actin levels were detected using mouse monoclonal anti‐β‐actin antibody (Proteintech, Cat. No. 66009–1) diluted 1:5000 in 5% milk in 1X TBST. Progerin levels were detected using rabbit polyclonal anti‐LaminA/C antibody (Abcam, Cat. No. ab108595) diluted 1:500 in 5% BSA in 1X TBST. Anti‐rabbit IgG‐HRP Cell Signaling, Cat. No. and Anti‐mouse IgG‐HRP secondary antibodies were diluted 1:10,000 in 5% BSA and 5% milk in TBST, respectively. Detection was done using the Clarity Western ECL Substrate (BioRad, Cat. No. 170–5061) and blots were imaged using the ChemiDoc MP Imaging System (BioRad, Cat. No. 12003154).

### 4.17 qPCR Primers

**Table:** 

Gene name	Fw	Re
Actin	CATGTACGTTGCTATCCAGGC	CTCCTTAATGTCACGCACGAT
Progerin	GCAACAAGTCCAATGAGGACCA	CATGATGCTGCAGTTCTGGGGGCTCTGGAC
p16	ATGGAGCCTTCGGCTGACT	GTAACTATTCGGTGCGTTGGG
p21	AGTATGCCGTCGTCTGTTCG	GACTGCAAGACAGCCGACAAG
IL‐1B	ATGATGGCTTATTACAGTGGCA	GTCGGAGATTCGTAGCTGGA
IL‐6	GAAAAAGATGGATGCTTCCA	AACTGGATCAGGACTTTTGT
hTERT	CCGATTGTGAACATGGACTACG	CACGCTGAACAGTGCCTTC
IL‐8	TAGCCAGGATCCACAAGTCC	GCTTCCACATGTCCTCACAA

### 4.18 Data Analysis

Data visualization software (GraphPad prism 10.0 software) was used for analysis. The measurement data were expressed as Mean ± Standard Error of Mean (Mean ± SEM). A comparison of measured variables between two groups was tested by Student's *t*‐test. Data between multiple groups were compared by one‐way or two‐way ANOVA.

## Author Contributions

J.P.C. and A.M. conceived the study and designed the experiments. W.Q. and A.M. performed the experiments. K.D.C. generated and purified the TERT circRNA and validated its integrity and ability to express telomerase. H.L. generated the TERT mRNAs used in the studies. T.K.C.N. helped to differentiate iPSCs into ECs. D.L.K. designed the codon optimality formula used to optimize the expression and increase the lifespan of the RNA constructs, and D.L.K. also supervised circRNA production and validation. W.Q. and A.M. generated the figures. W.Q., K.D.C., and A.M. wrote sections of the manuscript.

## Conflicts of Interest

J.P.C. is an inventor on patents owned by Stanford University and Houston Methodist Hospital related to the use of mRNA telomerase for the treatment of senescence and is a co‐founder of a company that will commercialize the telomerase technology. D.L.K. is a member of the scientific advisory board of the company mentioned above. Further, D.L.K. is an inventor on a patent owned by Houston Methodist Hospital related to the generation and use of circular RNA as a therapeutic platform.

## Supporting information


**Data S1.** **Figure S1:** TERT circRNA recovers nuclear morphology in the dose dependent manner. Representative images of nuclear staining with laminA for HGPS‐ECs upon treatments with 1 μg/mL TERT mRNA and different dosages of TERT circRNA, including 1, 0.5, 0.3, and 0.1 μg/mL, are shown (scale bar: 75 μm). Quantification of the percentage of lobulated nuclei 3–4 days after transfection. More than 200nuclei were quantified in a blinded manner (*n* = 4). Data between multiple groups were compared by one‐way ANOVA. Data Results were considered statistically significant with *p* < 0.05(*). Each dot represents one field from at least three replicates.
**Figure S2:** TERT circRNA persistently maintains telomere length.
Representative images of quantitative fluorescence in situ hybridization (q‐FISH) for non‐HGPS‐ECs, HGPS‐ECs, and HGPS‐ECs treated with TERT mRNA or TERT circRNA are shown (scale bar: 10 μm). To examine each image and determine changes, telomere and DAPI intensities were obtained, and telomere probe intensities were normalized to the DAPI signal for each individual nucleus. Quantification of q‐FISH shows telomere length in HGPS‐ECs treated with TERT circRNA or TERT mRNA, 28 days after a one‐time treatment. Data between multiple groups were compared by one‐way ANOVA. Data Results were considered statistically significant with *p* < 0.001(***), and *p* < 0.0001(****). Each dot represents one cell from at least three replicates.
**Figure S3:** Validating protein expression from optimal TERT RNA. A. Circularization and purification of TERT circRNA. 300 ng of RNA column‐purified in vitro transcription reaction (IVT), circularization reaction without RNase R (Circ‐RR) and with RNase R (Circ+RR) were resolved using 1.2% RNA FlashGel in denaturing conditions. B. Validation of RNA integrity. TapeStation gel electrophoresis depicting the sizes of TERT mRNA and TERT circRNA (left) and their corresponding absorbance traces (right). C. TERT protein expression in U2OS cells. Representative western blot of protein lysates collected 24 h post transfection from U2OS cells (1 × 10^6^ cells) transfected with 1 μg/mL of TERT RNA or TERT circRNA. Samples were probed with anti‐TERT (top blot) and anti‐β‐actin (bottom blot) antibodies. The theoretical MW for TERT protein expressed from the TERT mRNA or TERT circRNA is ~127.8 kDa.

## Data Availability

Data sharing is not applicable to this article as no new data were created or analyzed in this study.
