# Supplementary material for: Circular RNA Telomerase Reverses Endothelial Senescence in Progeria
Source: Aging Cell. 2025 Feb 23;24(6):e70021. doi: 10.1111/acel.70021 (PMC12151875; doi:10.1111/acel.70021)

Figures

Circular RNA telomerase reverses endothelial senescence in Progeria

Weifeng Qin, Kathrina D. Castillo, Hongye Li, Thi Kim Cuc Nguyen, Daniel L. Kiss, John P. Cooke*, Anahita Mojiri*

Center for Cardiovascular Regeneration & Center for RNA Therapeutics, Department of Cardiovascular Sciences, Houston Methodist Research Institute, 6670 Bertner Ave., R10-South, Houston, TX 77030, USA.

Figure 1:

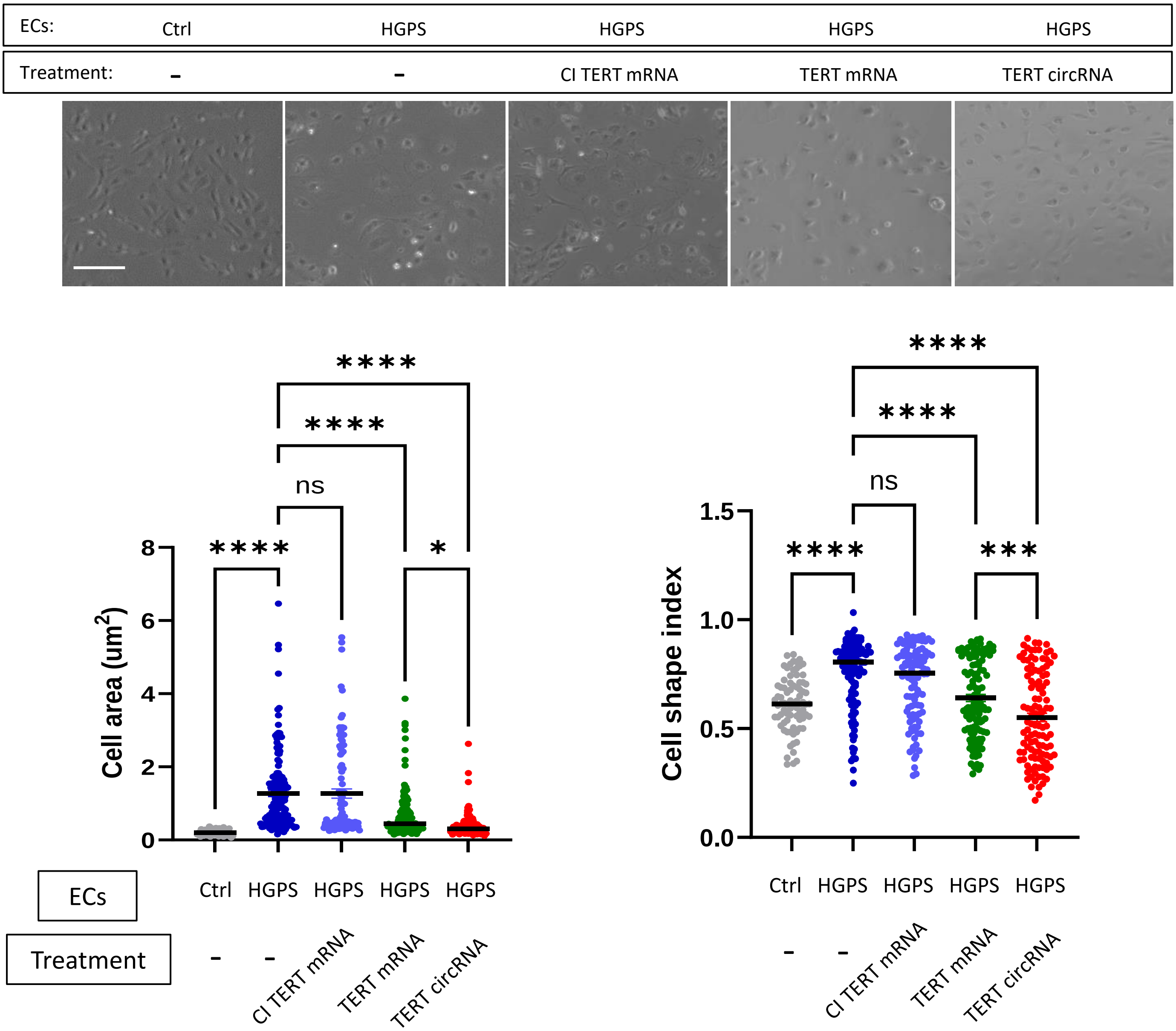

ECs: Ctrl HGPS HGPS HGPS HGPS
Treatment: – – CI TERT mRNA TERT mRNA TERT circRNA
Cell area (um2)
8
6
4
2
0
****
****
ns
****
*
Cell shape index
1.5
1.0
0.5
0.0
****
****
ns
****
***
ECs
Treatment
Ctrl HGPS HGPS HGPS HGPS
- - CI TERT mRNA TERT mRNA TERT circRNA
Ctrl HGPS HGPS HGPS HGPS
- - CI TERT mRNA TERT mRNA TERT circRNA

Figure 2:

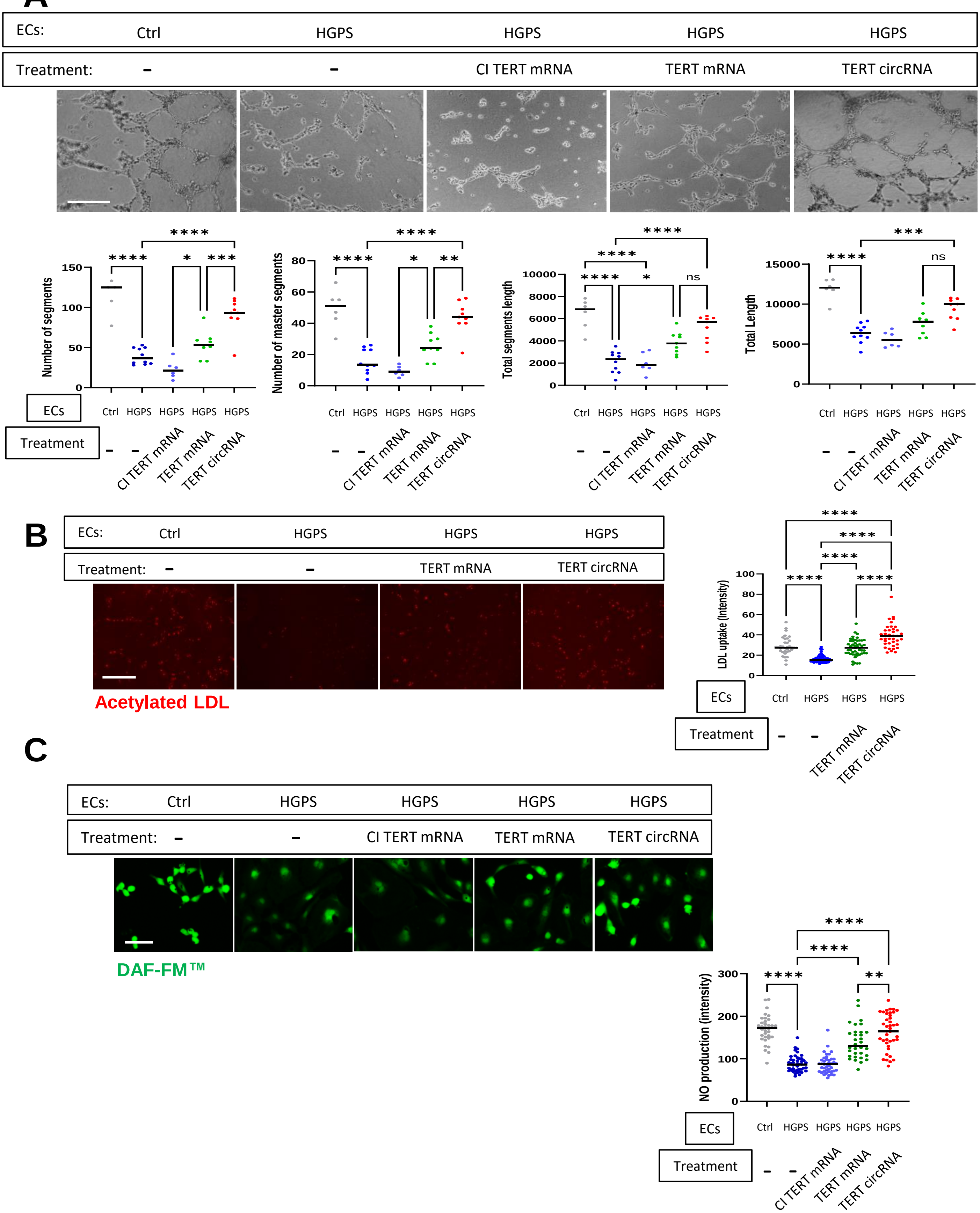
A
ECs: Ctrl HGPS HGPS HGPS HGPS
Treatment: – – CI TERT mRNA TERT mRNA TERT circRNA
Number of segments
Number of master segments
Total segments length
Total Length
ECs
Treatment
Ctrl HGPS HGPS HGPS HGPS
– – CI TERT mRNA TERT mRNA TERT circRNA
****
*
***
**
ns
B
ECs: Ctrl HGPS HGPS HGPS
Treatment: – – TERT mRNA TERT circRNA
Acetylated LDL
LDL uptake (Intensity)
Ctrl HGPS HGPS HGPS
– – TERT mRNA TERT circRNA
C
ECs: Ctrl HGPS HGPS HGPS HGPS
Treatment: – – CI TERT mRNA TERT mRNA TERT circRNA
DAF-FM™
NO production (intensity)
Ctrl HGPS HGPS HGPS HGPS
– – CI TERT mRNA TERT mRNA TERT circRNA

Figure 3:

A Dose dependent treatment measured after 3 days

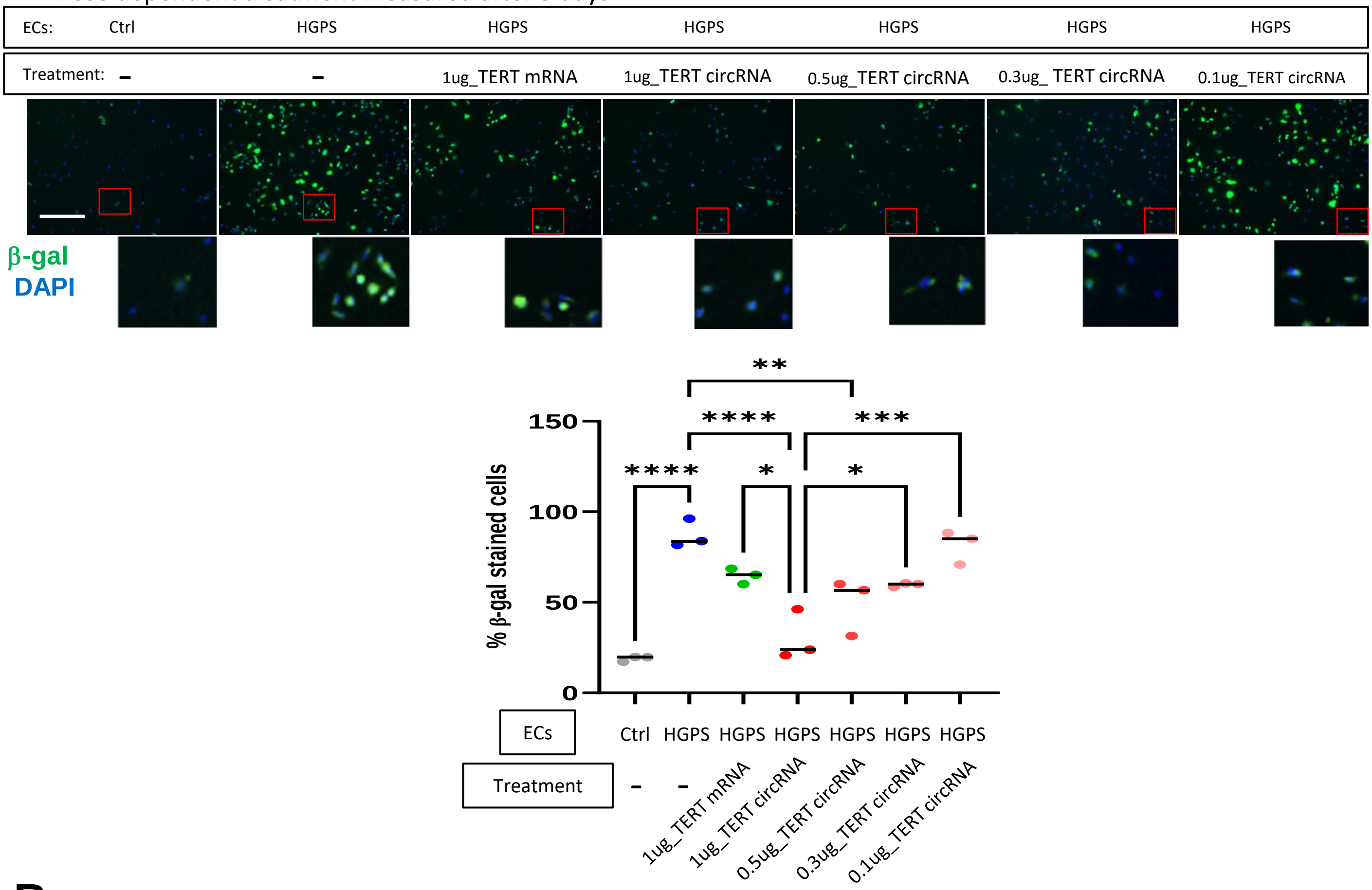

B

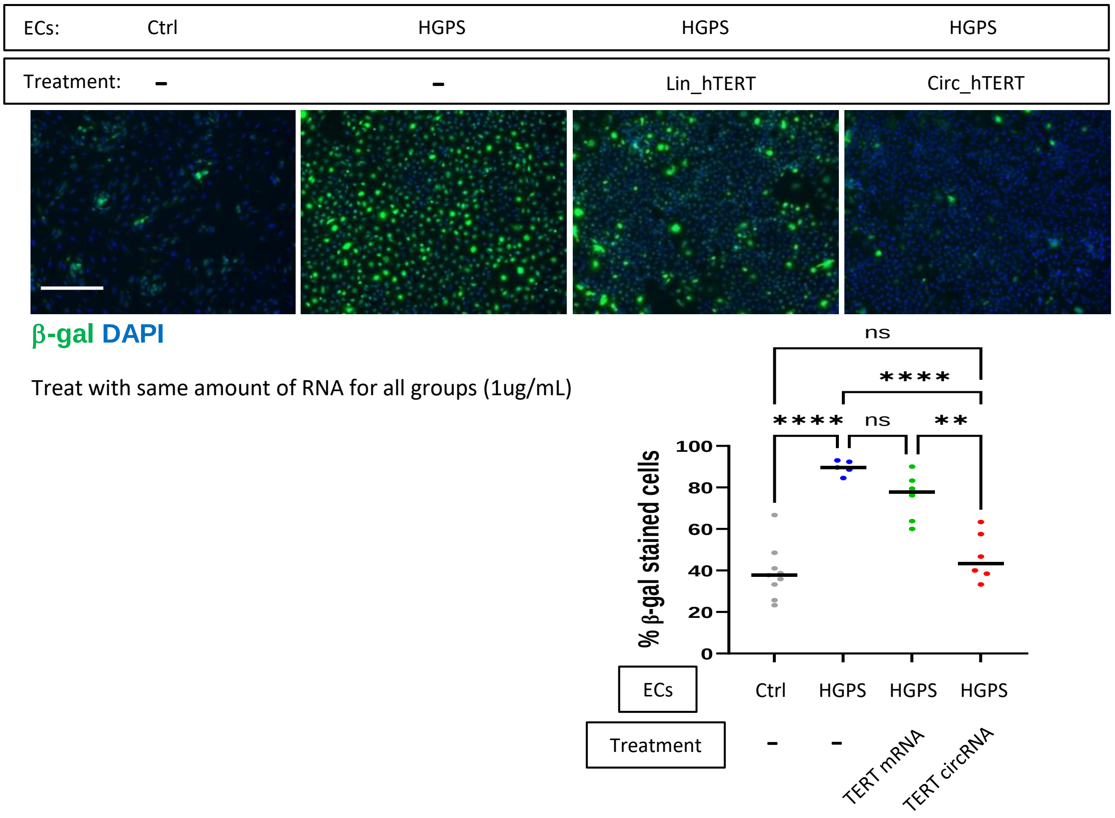

Figure 4:

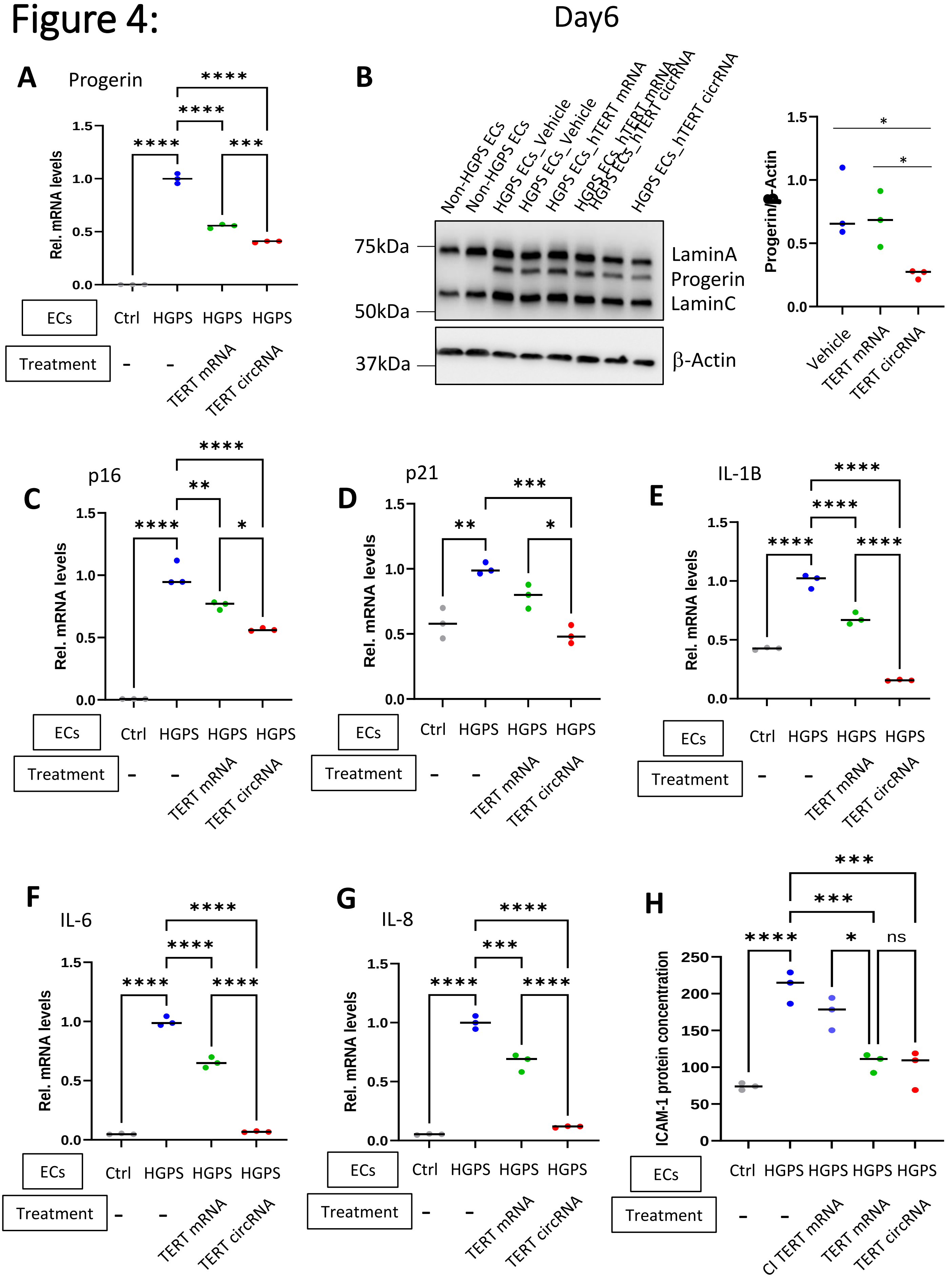

Day6
A
Progerin
Rel. mRNA levels
ECs
Ctrl HGPS HGPS HGPS
Treatment
TERT mRNA
TERT circRNA
B
Non-HGPS ECs
Non-HGPS ECs
HGPS ECs_Vehicle
HGPS ECs_Vehicle
HGPS ECs_hTERT mRNA
HGPS ECs_hTERT mRNA
HGPS ECs_hTERT circRNA
HGPS ECs_hTERT cicrRNA
75kDa
50kDa
37kDa
LaminA
Progerin
LaminC
β-Actin
Progerin/β-Actin
Vehicle
TERT mRNA
TERT circRNA
C
p16
D
p21
E
IL-1B
F
IL-6
G
IL-8
H
ICAM-1 protein concentration
CI TERT mRNA

Figure 5:

A

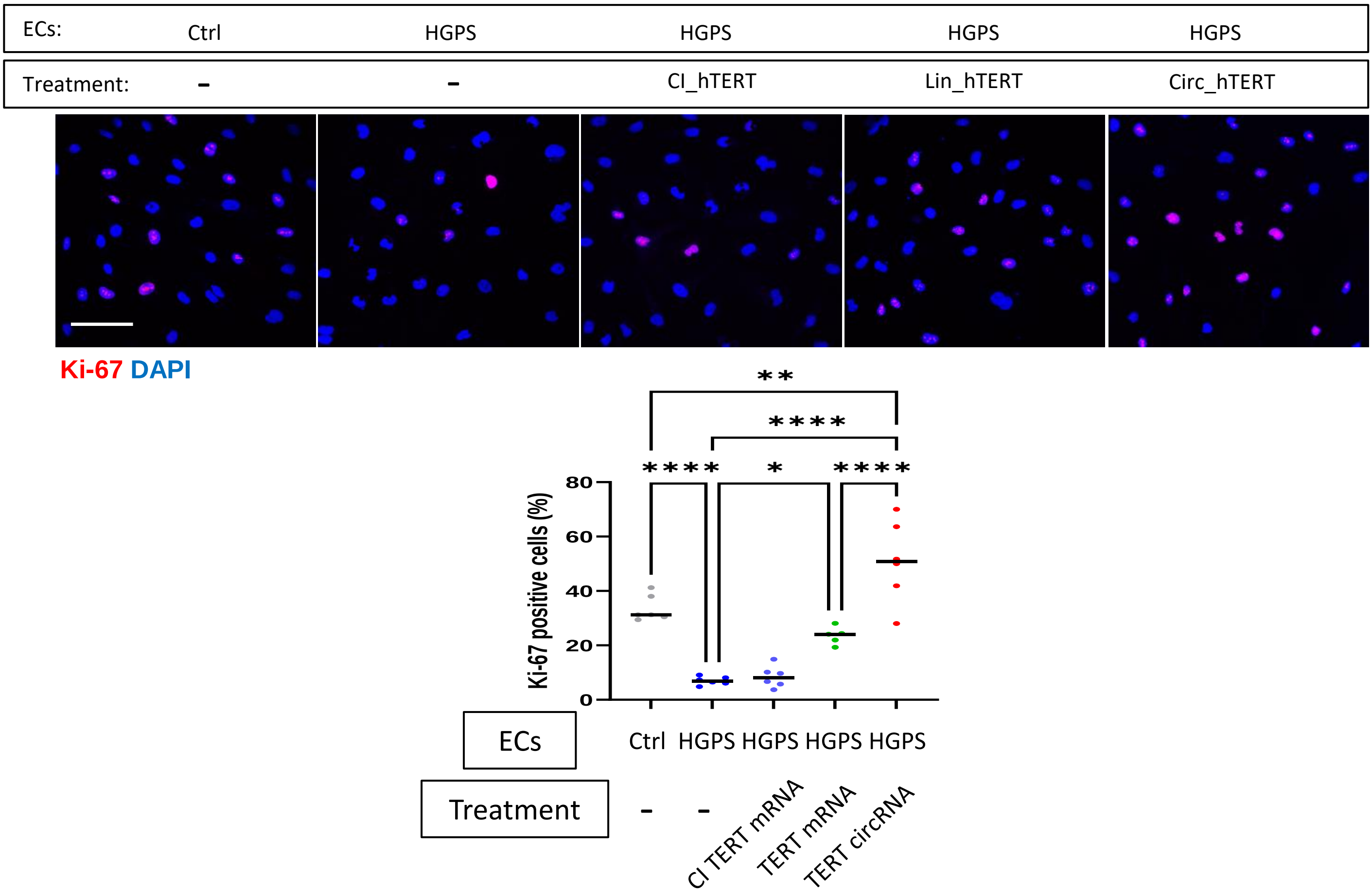

ECs:
Ctrl
HGPS
HGPS
HGPS
HGPS
Treatment:
–
–
Cl_hTERT
Lin_hTERT
Circ_hTERT
Ki-67 DAPI
**
****
****
*
****
Ki-67 positive cells (%)
80
60
40
20
0
ECs
Ctrl HGPS HGPS HGPS HGPS
Treatment
–
–
CI TERT mRNA
TERT mRNA
TERT circRNA

B

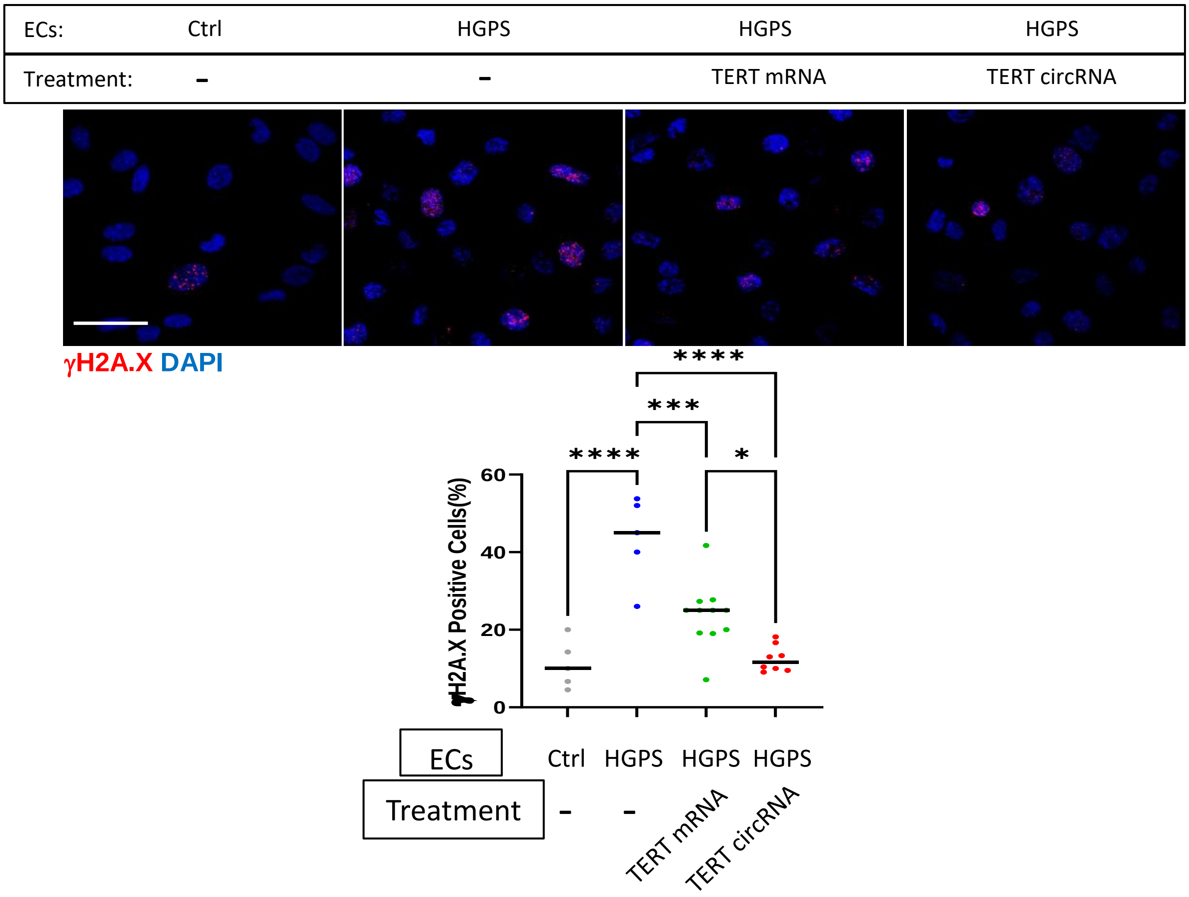

ECs:
Ctrl
HGPS
HGPS
HGPS
Treatment:
–
–
TERT mRNA
TERT circRNA
γH2A.X DAPI
****
***
****
*
γH2A.X Positive Cells(%)
60
40
20
0
ECs
Ctrl HGPS HGPS HGPS
Treatment
–
–
TERT mRNA
TERT circRNA

Figure 6:

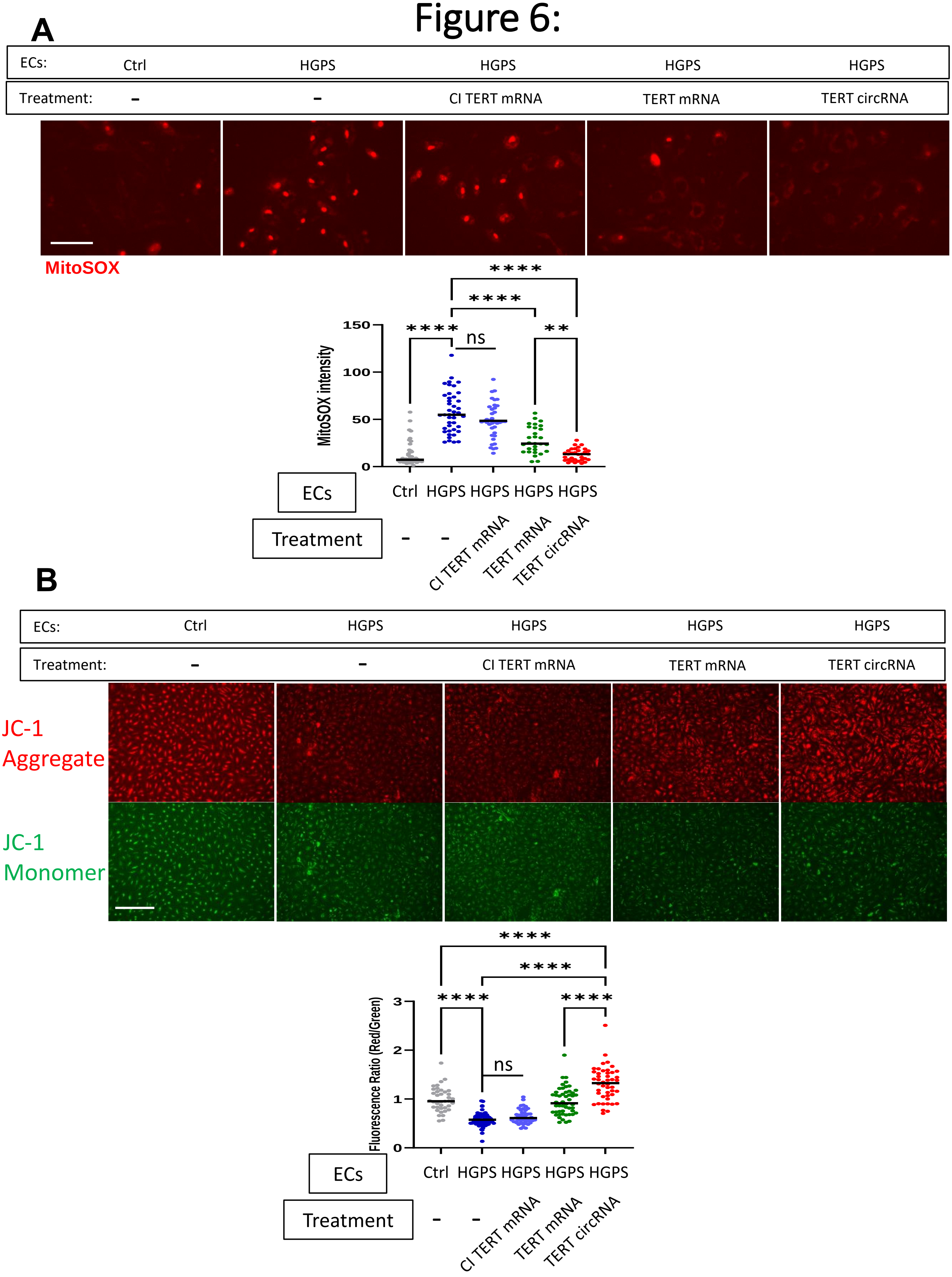
A
ECs: Ctrl HGPS HGPS HGPS HGPS
Treatment: - - CI TERT mRNA TERT mRNA TERT circRNA
MitoSOX
MitoSOX intensity
150
100
50
0
****
ns
**
ECs
Ctrl HGPS HGPS HGPS HGPS
Treatment
- - CI TERT mRNA TERT mRNA TERT circRNA
B
ECs: Ctrl HGPS HGPS HGPS HGPS
Treatment: - - CI TERT mRNA TERT mRNA TERT circRNA
JC-1 Aggregate
JC-1 Monomer
Fluorescence Ratio (Red/Green)
3
2
1
0
****
ns
ECs
Ctrl HGPS HGPS HGPS HGPS
Treatment
- - CI TERT mRNA TERT mRNA TERT circRNA

Figure 7:

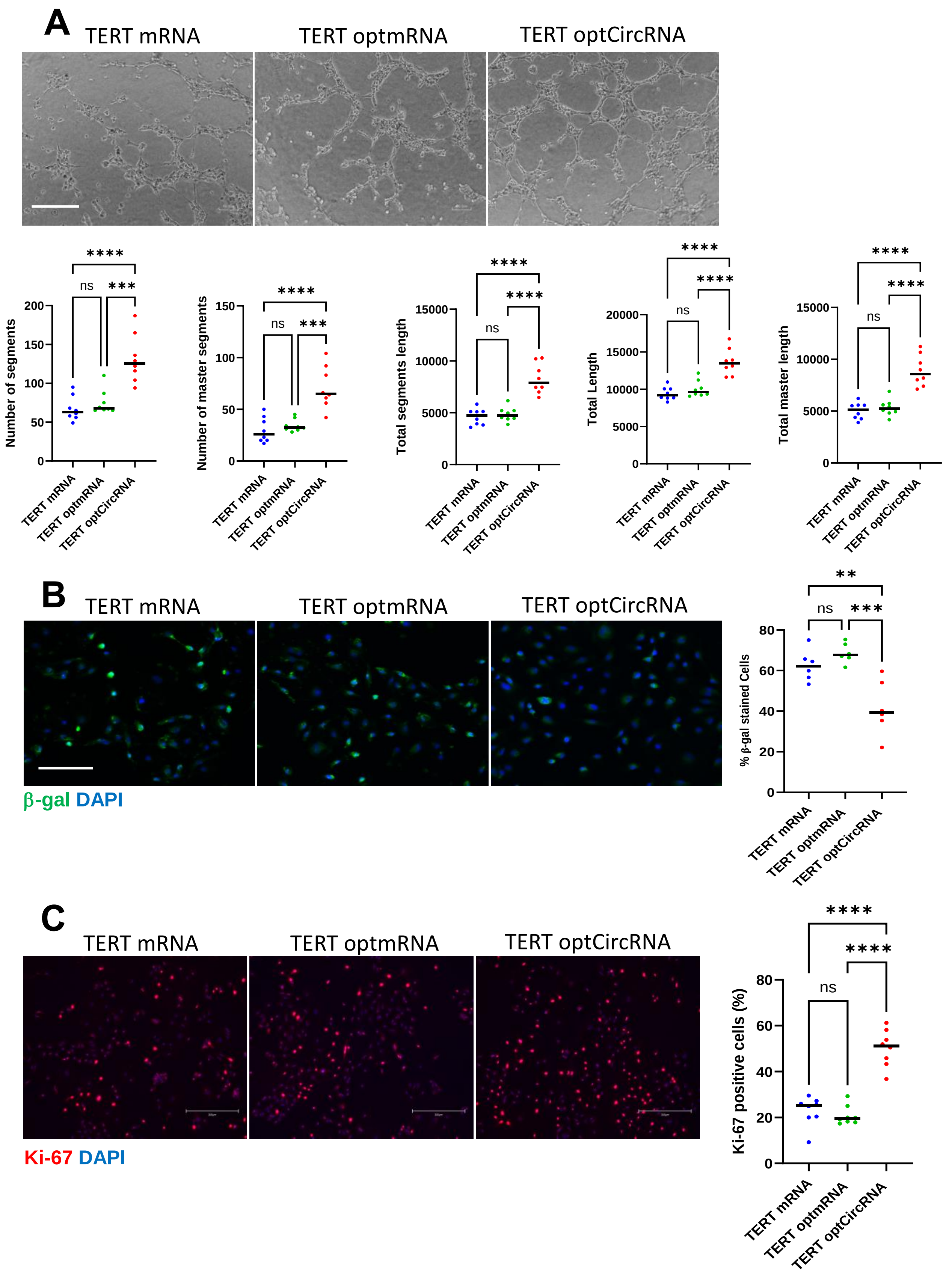
A
TERT mRNA
TERT optmRNA
TERT optCircRNA
Number of segments
Number of master segments
Total segments length
Total Length
Total master length
ns
****
***
B
TERT mRNA
TERT optmRNA
TERT optCircRNA
β-gal DAPI
% β-gal stained Cells
**
C
TERT mRNA
TERT optmRNA
TERT optCircRNA
Ki-67 DAPI
Ki-67 positive cells (%)

Figure 8:

A

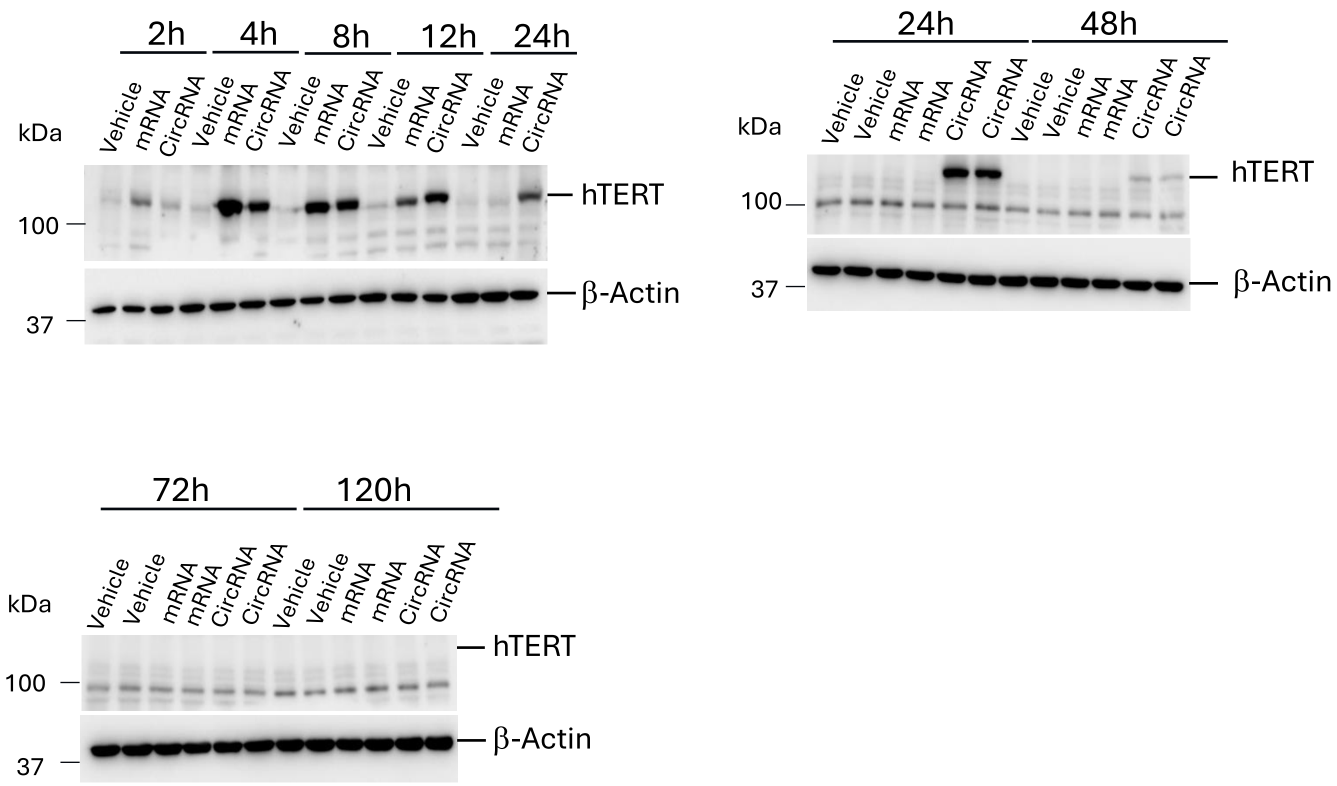

2h
4h
8h
12h
24h
Vehicle
mRNA
CircRNA
kDa
100
37
hTERT
β-Actin
24h
48h
72h
120h

B

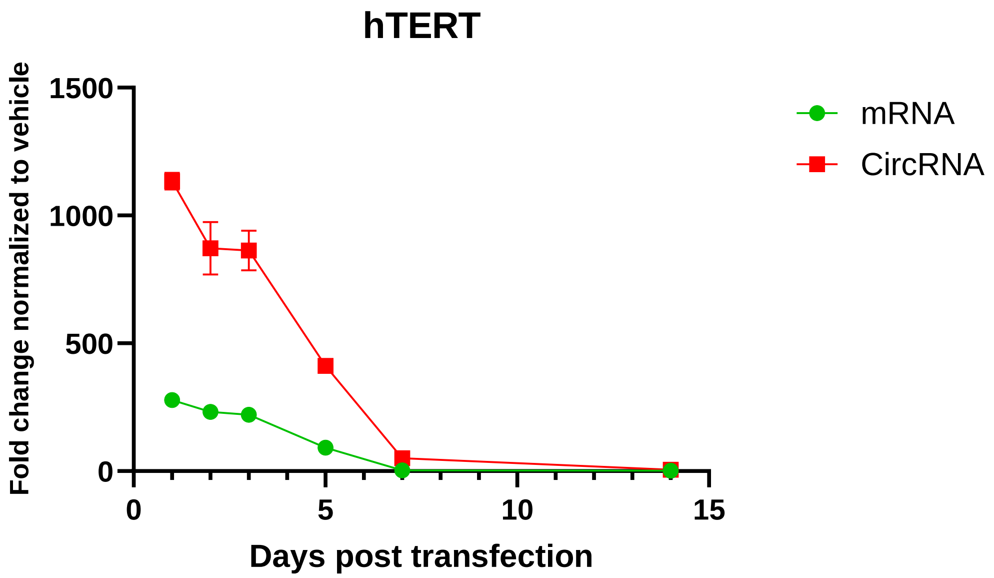

hTERT
Fold change normalized to vehicle
1500
1000
500
0
5
10
15
Days post transfection
mRNA
CircRNA

Supplement Figure 1:

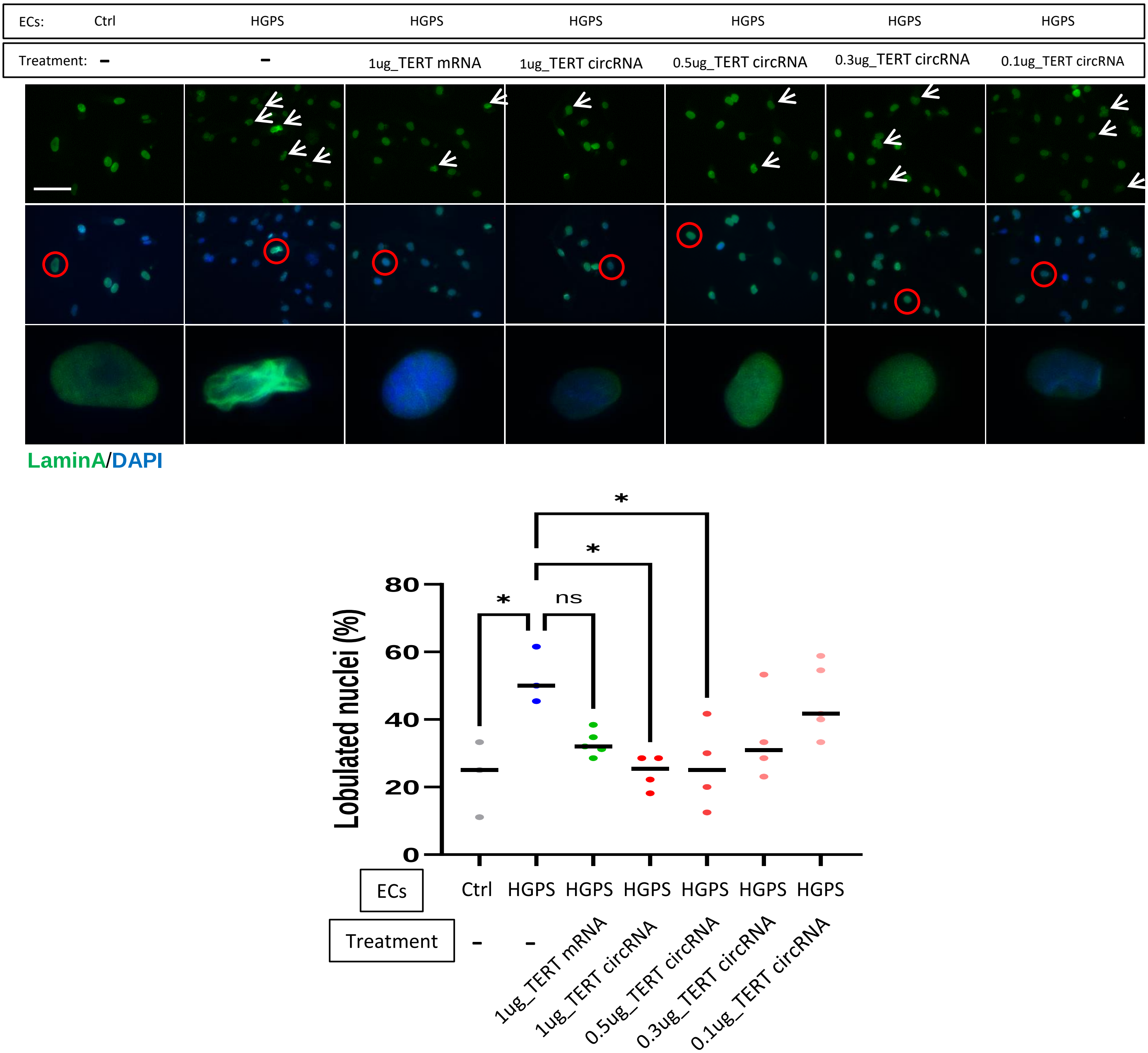

Supplement Figure 2:

Telomere length measurement 28 days after transfection

ECs:	Ctrl	HGPS	HGPS	HGPS
Treatment:	–	–	TERT mRNA	TERT circRNA

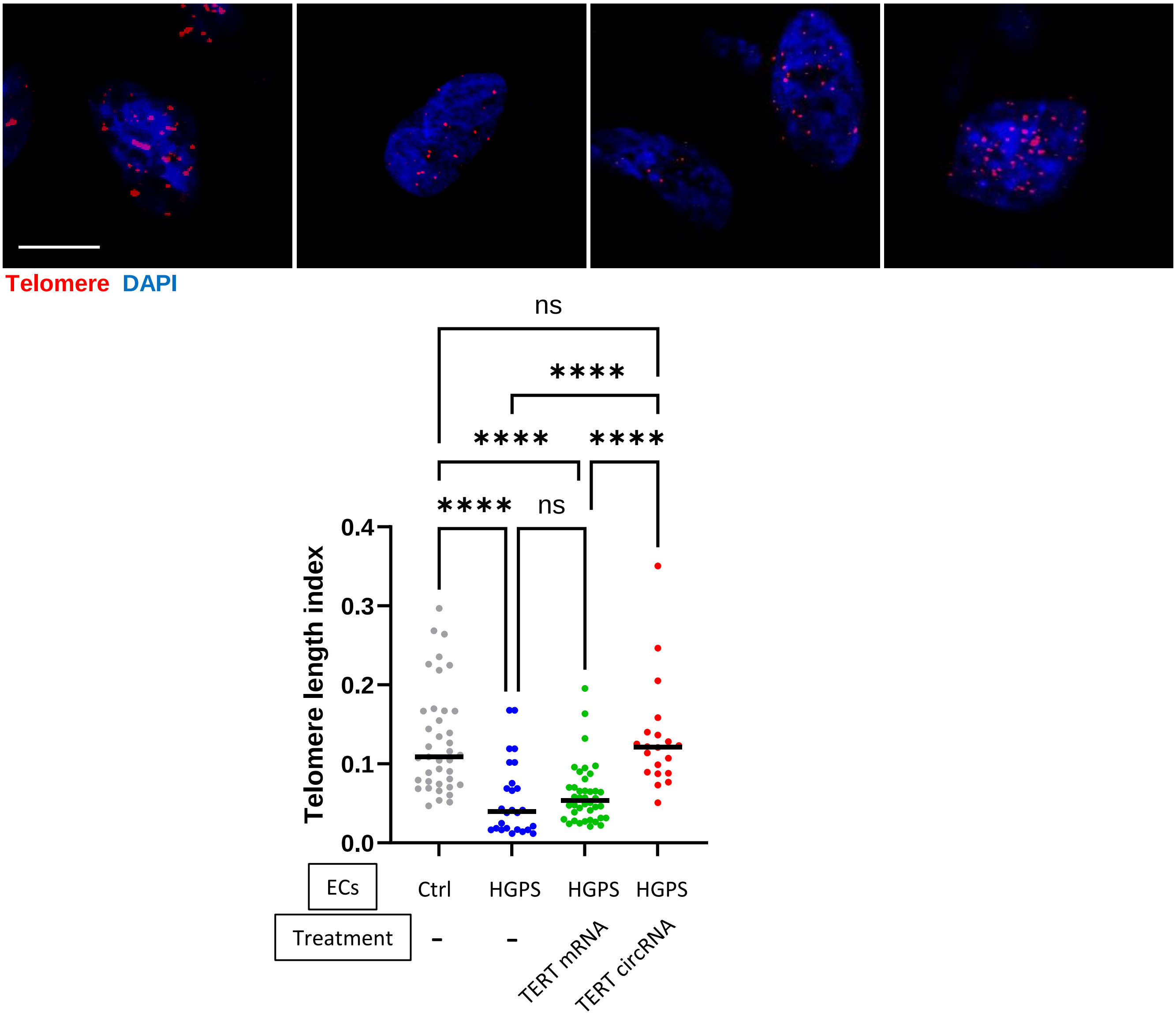

Supplement Figure 3:

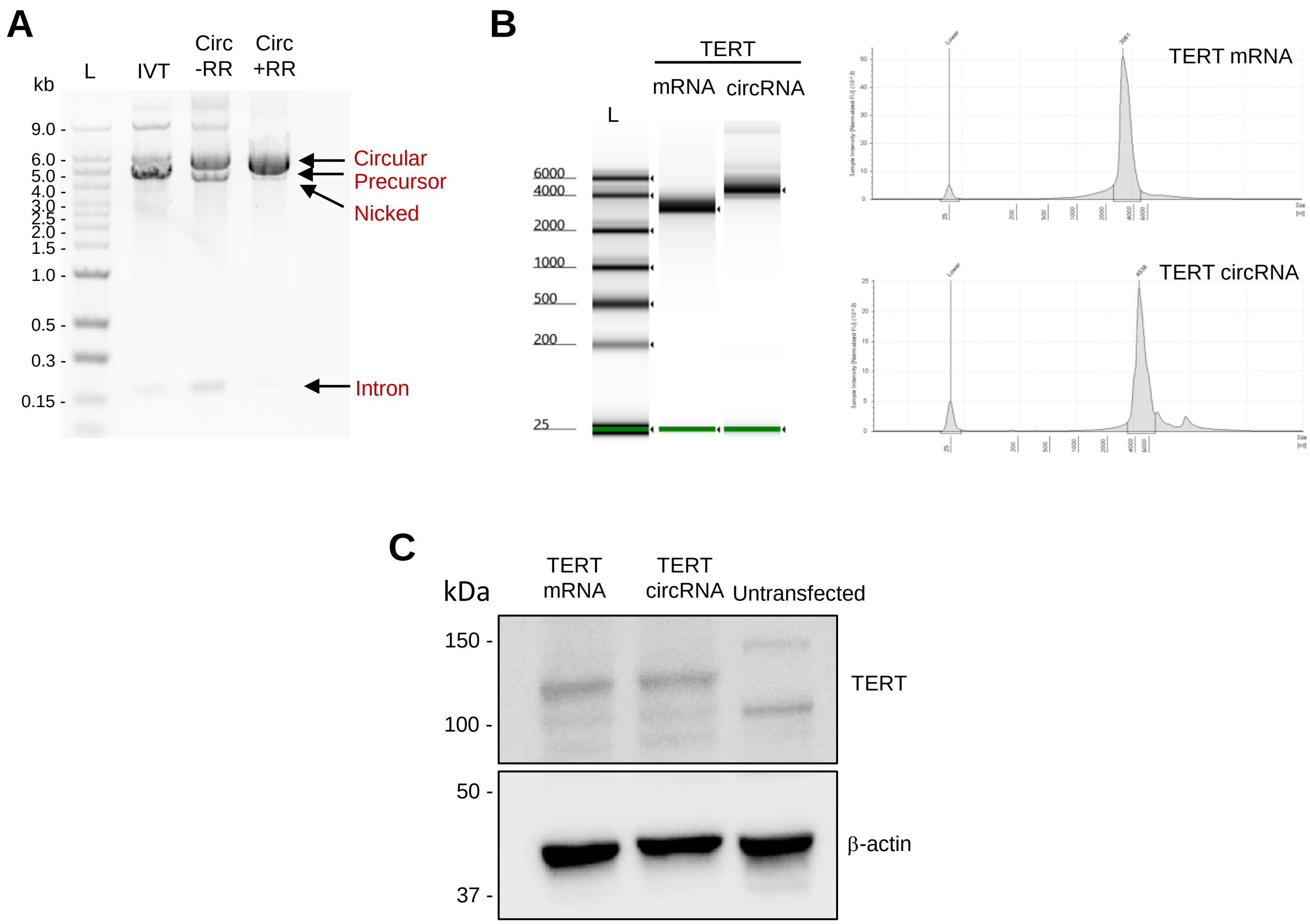

Supplement: Supplementary file 1 — Data S1. Figure S1: TERT circRNA recovers nuclear morphology in the dose dependent manner. Representative images of nuclear staining with laminA for HGPS‐ECs upon treatments with 1 μg/mL TERT mRNA and different dosages of TERT circRNA, including 1, 0.5, 0.3, and 0.1 μg/mL, are shown (scale bar: 75 μm). Quantification of the percentage of lobulated nuclei 3–4 days after transfection. More than 200nuclei were quantified in a blinded manner (n = 4). Data between multiple groups were compared by one‐way ANOVA. Data Results were considered statistically significant with p < 0.05(*). Each dot represents one field from at least three replicates. Figure S2: TERT circRNA persistently maintains telomere length. Representative images of quantitative fluorescence in situ hybridization (q‐FISH) for non‐HGPS‐ECs, HGPS‐ECs, and HGPS‐ECs treated with TERT mRNA or TERT circRNA are shown (scale bar: 10 μm). To examine each image and determine changes, telomere and DAPI intensities were obtained, and telomere probe intensities were normalized to the DAPI signal for each individual nucleus. Quantification of q‐FISH shows telomere length in HGPS‐ECs treated with TERT circRNA or TERT mRNA, 28 days after a one‐time treatment. Data between multiple groups were compared by one‐way ANOVA. Data Results were considered statistically significant with p < 0.001(***), and p < 0.0001(****). Each dot represents one cell from at least three replicates. Figure S3: Validating protein expression from optimal TERT RNA. A. Circularization and purification of TERT circRNA. 300 ng of RNA column‐purified in vitro transcription reaction (IVT), circularization reaction without RNase R (Circ‐RR) and with RNase R (Circ+RR) were resolved using 1.2% RNA FlashGel in denaturing conditions. B. Validation of RNA integrity. TapeStation gel electrophoresis depicting the sizes of TERT mRNA and TERT circRNA (left) and their corresponding absorbance traces (right). C. TERT protein expression in U2OS cells. Representative [file ACEL-24-e70021-s001.pdf]
